# Supplementary material for: INSIHGT: an accessible multi-scale, multi-modal 3D spatial biology platform
Source: Nat Commun. 2024 Dec 30;15:10888. doi: 10.1038/s41467-024-55248-0 (PMC11685604; doi:10.1038/s41467-024-55248-0)
Supplement: Supplementary file 1 — Supplementary Information [file 41467_2024_55248_MOESM1_ESM.pdf]

Supplementary Information

INSIHGT: An accessible multi-scale, multi-modal 3D spatial biology platform

Supplementary Texts

Weakly coordinating superchaotropes as non-denaturing protein solubilizers

For proteins, the significant role of intramolecular interactions in intermolecular interactions complicates the picture. Ideally, intermolecular interactions were selectively disturbed for solubilization, with minimal disturbance on intramolecular interactions to prevent denaturation. We reasoned that if the interactions between the native solvent (i.e., water) and proteins are promoted, the intramolecular forces (e.g., hydrophobic interactions) of proteins that evolved in an aqueous environment will help maintain their current folded state. This contrasts with agents that have significant affinities towards the proteins, which may compete against the intramolecular forces and disrupt their folding, such as with detergents [1] and strong hydrogen-bonding agents like urea [2]. Hence, an ideal protein solubilizer is water soluble yet poorly coordinating with both water and proteins. The ideal weakly-coordinating superchaotropes have low charge-to-volume ratios and lack hydrophobic groups to avoid affinities towards protein hydrophobic surfaces. We thus screened the literature for counter anions used in the isolation of extremely electrophilic species for crystallography [3] or as conjugate bases of superacids, which include perchlorate, perrhenate, and dodecaborates.

Physicochemical properties of *closo*-dodecahydrododecaborate

The *closo*-dodecahydrododecaborate $[B_{12}H_{12}]^{2-}$ was discovered in the 1950s and stands out as a remarkably stable member of boron cluster compounds and boron hydrides. It can withstand sustained dry heating, strong acids and alkalis, and diverse reactive organic or inorganic chemicals. Its chemical inertness has been attributed to the 3D aromaticity of the ion - a Hückel's rule generalization in planar aromatic compounds. Apart from chemical inertness, $[B_{12}H_{12}]^{2-}$ is also poorly coordinating, exemplified by the super-acidic character of its Brønsted acid $H_2B_{12}H_{12}$ (pK_a = 28) [4] and perhaps by the stabilization of the conjugate base by delocalizing the two electrons over a large surface area. The combined weak ionic and poorly coordinating properties lead to easy solubilization of the $[B_{12}H_{12}]^{2-}$ ion in diverse solvents, lending the proposal for it to be used for nuclear waste treatment by extracting charged ions into water-immiscible organic solvents. The chemical and coordinative inertness of $[B_{12}H_{12}]^{2-}$ is also directly related to its biological inertness, with an oral lethal dose (LD_{50}) for rats at > 7,500 mg/kg. Hence, the proposed biological applications for $[B_{12}H_{12}]^{2-}$ and its ^{10}B isotopic derivatives were largely related to boron neutron capture therapy (BNCT) *in vivo*. The utility of $[B_{12}H_{12}]^{2-}$ *in vitro* has been scarcely explored. The available solution characteristics of $[B_{12}H_{12}]^{2-}$ is summarized in **Supplementary Table 5**.

Interactions of $[B_{12}H_{12}]^{2-}$ with other molecular species

The pair correlation function of $[B_{12}H_{12}]^{2-}$ ion in water has been obtained by simulation. It showed concentric rings of water molecules on top of the hydrogen atoms, with an average number of water molecules forming ≈10 bridging O–H···H–B dihydrogen bonds (DHB) [5], with a simulated DHB length of 1.76 Å [6] with an estimated bond strength of 7.15 kcal mol^{-1} [7] (*ca.* O–H···O hydrogen bond length in water: 1.97 Å, bond strength 5.0 kcal mol^{-1}). Simulated translational diffusion coefficients of the $[B_{12}H_{12}]^{2-}$ ion in water is $(1.8 \pm 0.2) \times 10^{-9}$ m^2 s^{-1}.

Apart from forming DHBs, $[B_{12}H_{12}]^{2-}$ do have some coordinating activity as per evidence obtained in crystalline solid state [8] and has been shown to interact with adenine directly in aqueous solutions. [9]

Interestingly, at concentrations up to 1.5mM, $[B_{12}H_{12}]^{2-}$ does not disrupt the structure of bovine serum albumin (BSA) at all in 0.1M $NaHCO_3$, does not show any significant interactions with BSA or affect the hydrodynamic radii of BSA even at near 1,000-fold molar excess. It also did not affect the thermal denaturing behaviour of BSA. [10]

INSIHGT Protocol

This protocol describes the INSIHGT process for staining fixed or permeabilized tissues.

Materials

PBSN (1× PBS with 0.02% w/v NaN_3)	Sodium dodecahydrododecaborate ($Na_2B_{12}H_{12}$)
Methanol	2-hydroxypropyl-γ-cyclodextrin (2HPγCD)
Dichloromethane	Nucleic acid probe (optional)
Sodium sulphite (Na_2SO_3, optional)	Sulfobutylether-β-cyclodextrin (SBEβCD, optional)

Buffers/Solutions

INSIHGT buffer A (2×): 0.5M $Na_2B_{12}H_{12}$ in PBSN

INSIHGT buffer B (1×): 0.25M 2HPγCD in PBSN

INSIHGT buffer C (1×): 0.25M SBEβCD in PBSN

Procedure overview

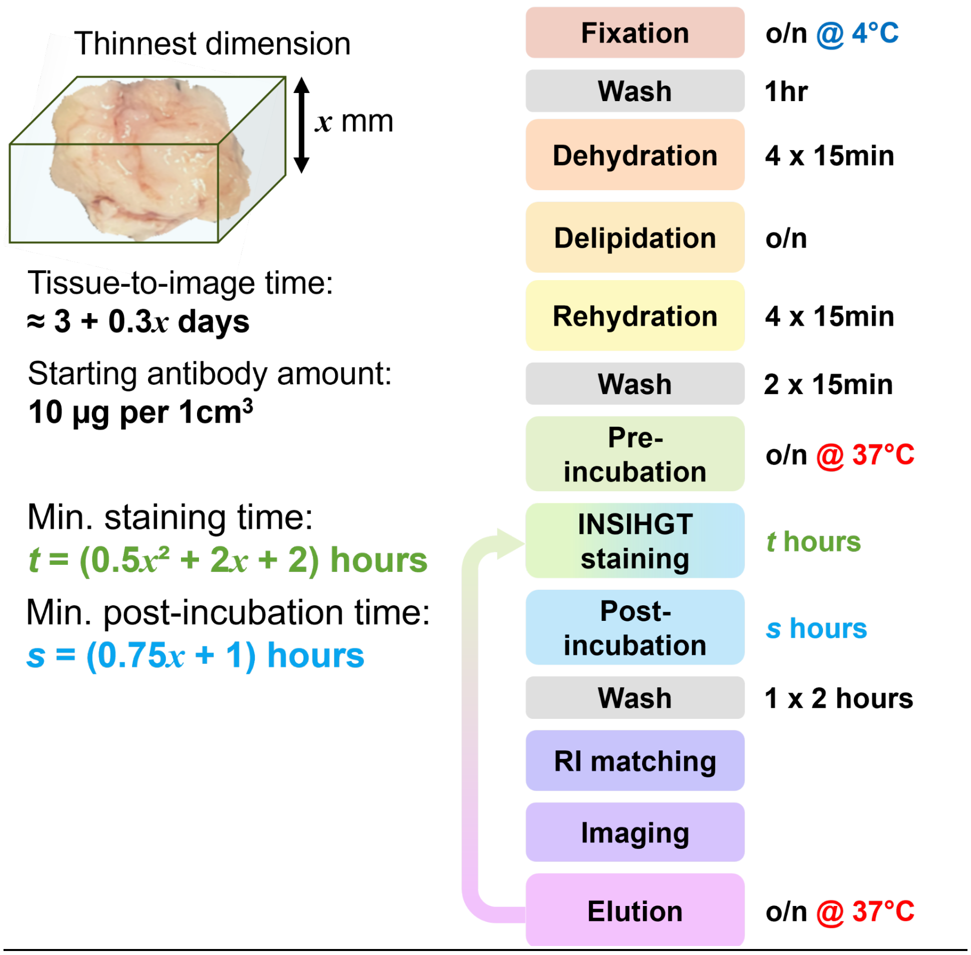

Procedure

The procedure below is illustrated for a 1 cm^3 tissue sample using BABB clearing but can be scaled as needed.

0. **Fixation:** Fix tissue overnight at 4°C with formaldehyde, glutaraldehyde or SHIELD-fixation. Wash in 5 ml PBSN for 1 hour at room temperature (r.t.) with shaking. For glutaraldehyde or SHIELD-fixation, quenching should be done appropriately.

1. **Dehydrate** by shaking incubation in 5 ml 50% v/v methanol/water, then 5 ml 100% methanol repeated 3 times, 15 min with shaking at r.t. for each step.

2. **Delipidate** by shaking incubation in 5 ml 2:1 v/v mixture of dichloromethane/methanol overnight at r.t. Use a glass or polypropylene container (e.g. microcentrifuge tubes), in a well-ventilated hood with adequate shaking.

3. **Rehydration:** Repeat the dehydration steps in reverse order, then 2 × 15 min washes in PBSN.

4. **Pre-Incubation:** Incubate in 2 ml 1× INSIHGT buffer A at 37°C overnight with shaking.

5. **Prepare nucleic acid probe (optional):** Prepare 200 nmol of the probe by mixing it with INSIHGT buffer C in a 1:1 v/v ratio.

6. **INSIHGT Staining:**

a. Place the tissue in a container that fits its size well.

b. Add enough 2× INSIHGT buffer A to around half-cover the tissue.

c. Add the desired amount of primary antibodies (start with 10µg each).

d. Add the prepared nucleic acid probe in INSIHGT buffer C (if desired).

e. Add corresponding fluorophore-labeled secondary antibody Fab fragments or nanobodies (in the same µg amount as the primary antibody used), or lectins if desired.

f. Add PBSN to make the final concentration of INSIHGT buffer A to 1x. The volume required will be equal to the volume added in step 6a - (total volume added in step 6c, 6d, and 6e).

g. Top up with 1x INSIHGT buffer A to just cover the entire tissue.

h. Incubate at r.t. for 3 days (or *t* hours, see below) with shaking. Protect from light.

7. **Post-incubation:** Incubate the stained tissue in 3 ml INSIHGT buffer B at r.t. overnight (or *s* hours, see below) with shaking. Place the tube horizontally to ensure adequate mixing.

8. **Wash**: Wash in 5 ml PBSN three times for 2 hours each at r.t., shaked horizontally.

9. **Refractive index matching** (optional): Example for BABB clearing:

- Repeat the dehydration steps.
- Prepare BABB: 1:2 v/v mix of benzyl alcohol / benzyl benzoate.
- Place tissue in 5ml BABB at r.t. overnight with shaking. Refresh BABB until tissue is clear.

10. **Imaging**: Confocal, light-sheet and two-photon microscopies are all compatible.

11. **Stripping** (optional): Strip tissue in 2 ml 1× INSIHGT buffer A + 0.1 M Na_2SO_3 at 37°C overnight with shaking. Wash twice with 2 ml 1× INSIHGT buffer at 37°C for 2 hours each.

12. **Repeat staining** (optional): Proceed to step 6 for the next round of staining.

<u>Notes</u>

- All incubation periods can be extended up to one month without harm.
- Tissues delipidated with methods other than CH_2Cl_2/Methanol are acceptable, except if the buffer contains Triton X-100 or triethylamine. Tween-20 is compatible.
- Test pretreatment buffer compatibility with INSIHGT. Mix 100µl of the buffer with 2× INSIHGT buffer A in a 1:1 ratio. If precipitation occurs, test each buffer component individually and exclude any that cause precipitation from your modified buffer.
- Avoid contact between 100% dichloromethane and plastics (except with polypropylene or PTFE tips or Eppendorf tubes). It dissolves plastics and makes the solution cloudy.
- The minimum staining time (t hours) in step 8 depends on the thinnest dimension (x, in mm) of the tissue. Calculate t as $0.5x^2 + 2x + 2$. The estimated minimum staining duration is employed throughout the manuscript to achieve protocol standardization.
- The minimum post-incubation time (s hours) in step 9 is $s = 0.75x + 1$. The estimated minimum post-incubation duration is employed throughout the manuscript to achieve protocol standardization.
- If staining is uniform, staining time can be reduced. Conversely, if penetration is inhomogeneous, ensure adequate amounts of antibodies have been added, and then extend the staining time by 1.5×. There is no need to adjust post-staining incubation and washing.
- Increasing antibody concentration in the staining solution by 1.5× can also enhance penetration homogeneity. Conversely, if staining is uniform and signal-to-noise is satisfactory, antibody concentration can be reduced.
- If using fluorophore-labeled whole IgG secondary antibodies, F(ab')2 fragments or multivalent streptavidins, perform steps 6–8 with primary antibodies only, then repeat for the secondary/tertiary agents starting from step 4. There is no need to post-fix primary antibodies.
- We recommend using INSIHGT with solvent-based tissue clearing (such as BABB, DBE, or ethyl cinnamate (ECi)) as they provide the fastest tissue delipidation options. Note that triethylamine should be replaced by triethanolamine in ECi clearing to avoid residual triethylamine precipitating with $[B_{12}H_{12}]^{2-}$. Similarly, for aqueous-based methods, any use of Triton X-100 should be replaced by other detergents that do not precipitate with $[B_{12}H_{12}]^{2-}$. Please refer to the above points on how to test buffer compatibility.
- To visualize endogenous fluorescence, staining them with anti-fluroescent protein

antibodies is the best solution. Alternatively, one can attempt clearing with (ECi) [61,62], with triethanolamine replacing triethylamine as pH adjusting agent, or dibenzyl ether clearing after tetrahydrofuran (THF) dehydration [63]. SHIELD-based protection with aqueous-based refractive index matching using PROTOS (refractive index matching solution; LifeCanvas Technilogies, Cambridge, MA, United States)[64] or OPTIClear[65] can also be used after omitting Triton X-100 in all the buffers.

- After staining, the sample can be stored in PBSN at 4°C or in BABB at r.t. Indefinitely.
- All equations for the minimum staining time and post-incubation time are empirically determined and shall be applicable to various tissue types, at times depending on the antigen densities, as discussed above. We suggest for extremely dense antigens or labelling (e.g., lectins, ubiquitous and highly expressed antigens), the user should optimize the staining duration and the amount of probe added using a bulk-then-cut-staining assessment as with our benchmarking experimental design (**Supplementary Fig. 4a**).

Graphical summary of INSIHGT protocol

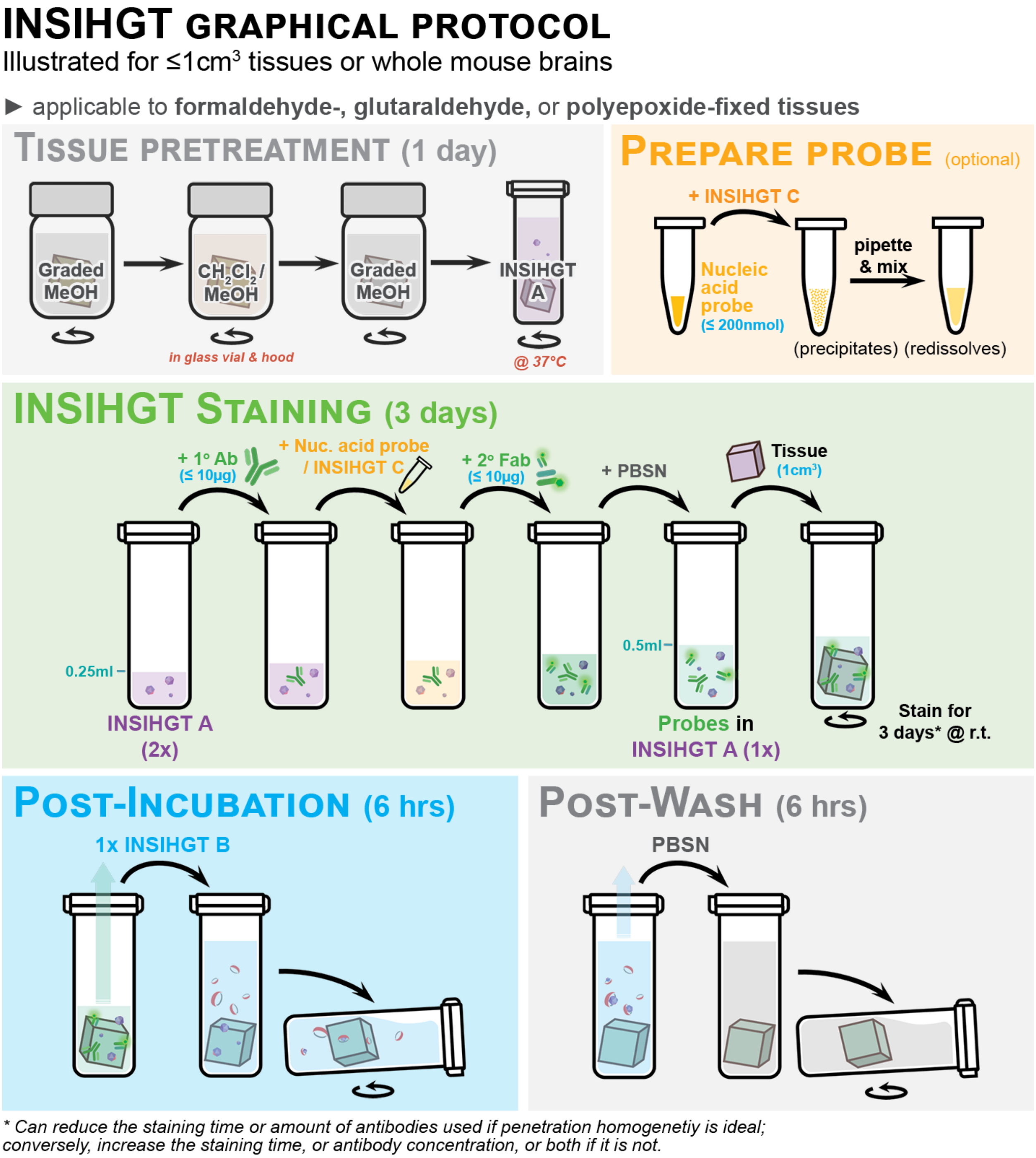

The above image was provided by Illumos Limited and reproduced here with permission.

Supplementary Figures

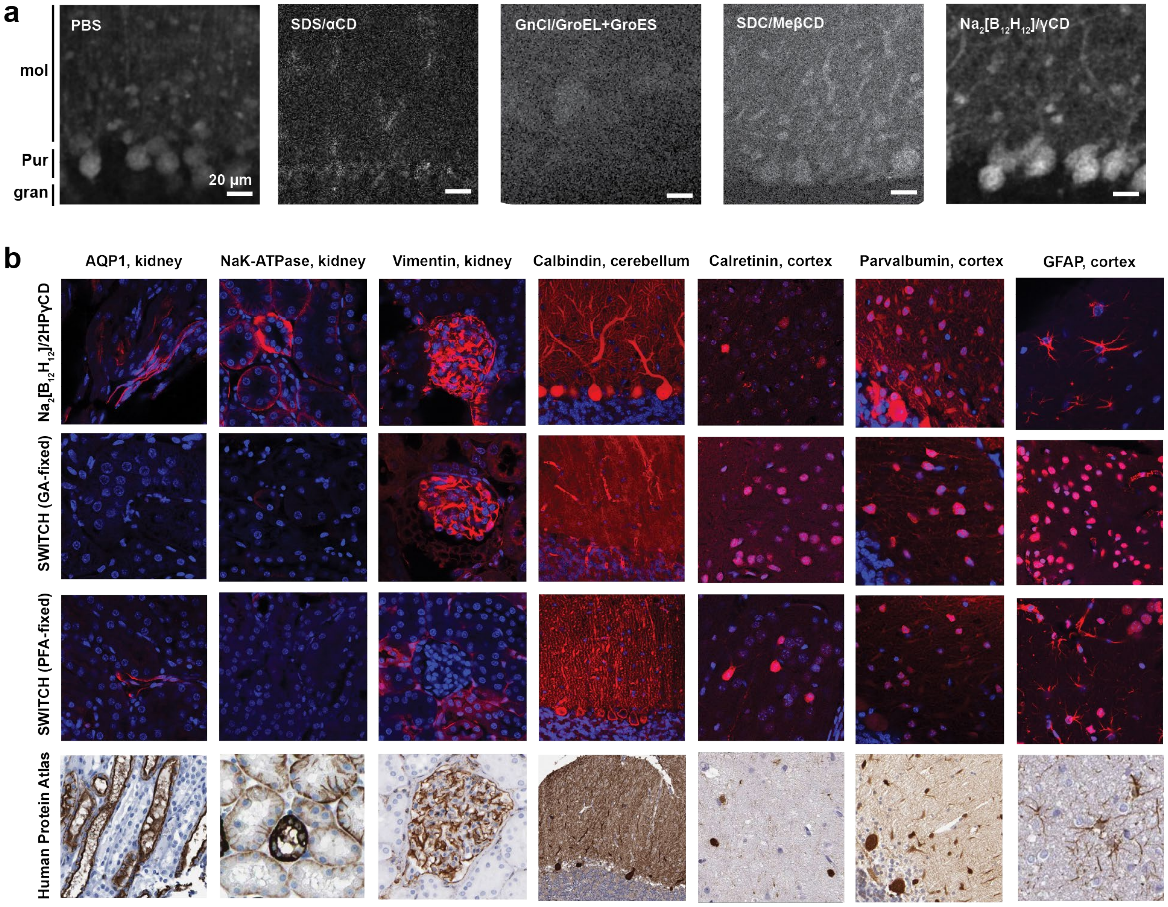

Supplementary Figure 1. Comparison of staining techniques in biological tissues. a Comparison of proposed antibody-antigen inhibition strategies and their recovery approaches. All stainings were performed in mouse cerebellum tissue for PVALB, with antibodies first co-incubated in a buffer containing an antibody-antigen interaction inhibitor followed by a measure to reinstate antibody-antigen binding. The samples were then cut midway on the longest axis and imaged at the cut face to evaluate the bulk-staining penetration depth and uniformity. PBS: staining for PVALB done in PBS as a control. SDS/αCD: inhibition with 10mM SDS, followed by its complexation with alpha-cyclodextrin (αCD) for recovery, GnCl/GroEL+GroES: inhibition with 6M guanidinium chloride (GnCl) followed by in situ refolding with GroEL and GroES molecular chaperones, SDC/MeβCD: inhibition with sodium deoxycholate (SDC) followed by its complexation with random methylated beta-cyclodextrin (MeβCD) for recovery, $Na_2[B_{12}H_{12}]$-γCD: inhibition with 0.125M sodium dodecahydro-closo-dodecaborate ($Na_2[B_{12}H_{12}]$) followed by its complexation with gamma-cyclodextrin (γCD) for recovery. Abbreviations: mol: molecular layer, pur: Purkinje layer, gran: granular layer. **b** Broader antibody compatibility and more specific staining was observed with the use of $Na_2[B_{12}H_{12}]$-2HPγCD system with conventional paraformaldehyde fixation (upper row), compared with the SWITCH-labelling method reported by Murray *et al* [11], regardless of whether the tissue was fixed as described using glutaraldehyde (GA-fixed) or paraformaldehyde (PFA-fixed, i.e., no SWITCH-fixation employed). Lower row: Human tissue immunostaining patterns from the Human Protein Atlas for comparison. DAPI staining for nuclei in blue, and antibody staining in red.

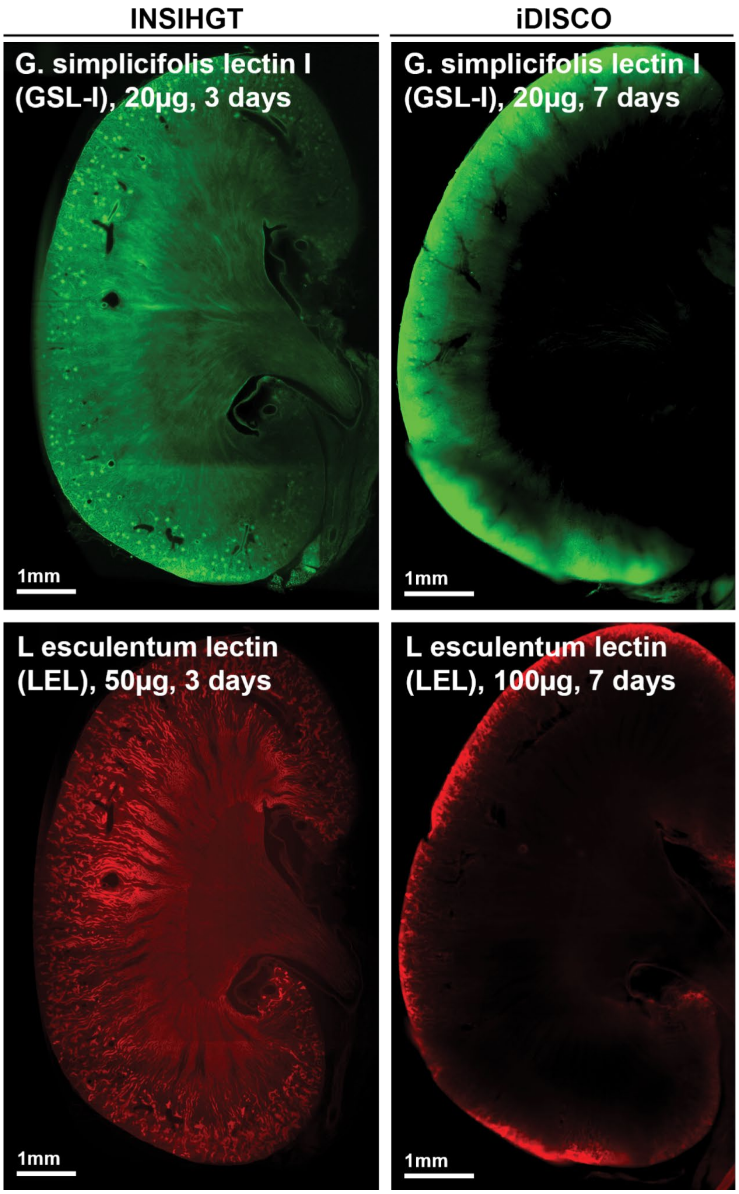

Supplementary Figure 2. Comparison of lectin staining results between INSIHGT and iDISCO. Lectin staining often results in a significant rimming pattern where only superficial layers are stained, as demonstrated via iDISCO staining of mouse kidney with GSL-I and LEL.

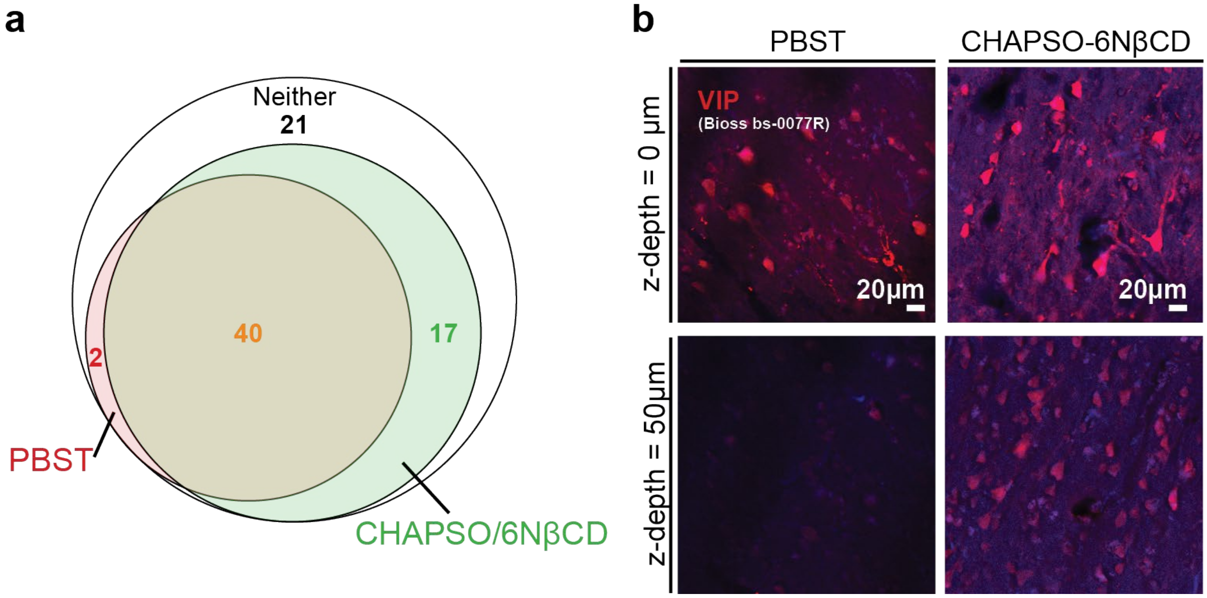

Supplementary Figure 3. Efficacy of CHAPSO/6βCD host-guest system in antibody penetration and staining depth in tissues. a The use of an optimized artificial chaperone host-guest system, CHAPSO/6NβCD, is compatible with 57 out of 80 validated commercial primary antibodies on human cancer tissue, human brain tissue, mouse kidney tissue and mouse brain tissue, compared to the use of PBS with 1% Triton X-100 (PBST) as the staining buffer. **b** Examples in the head-to-head staining penetration comparison using human brain tissue. DAPI signals were colored in blue, while antibody signals were colored in red.

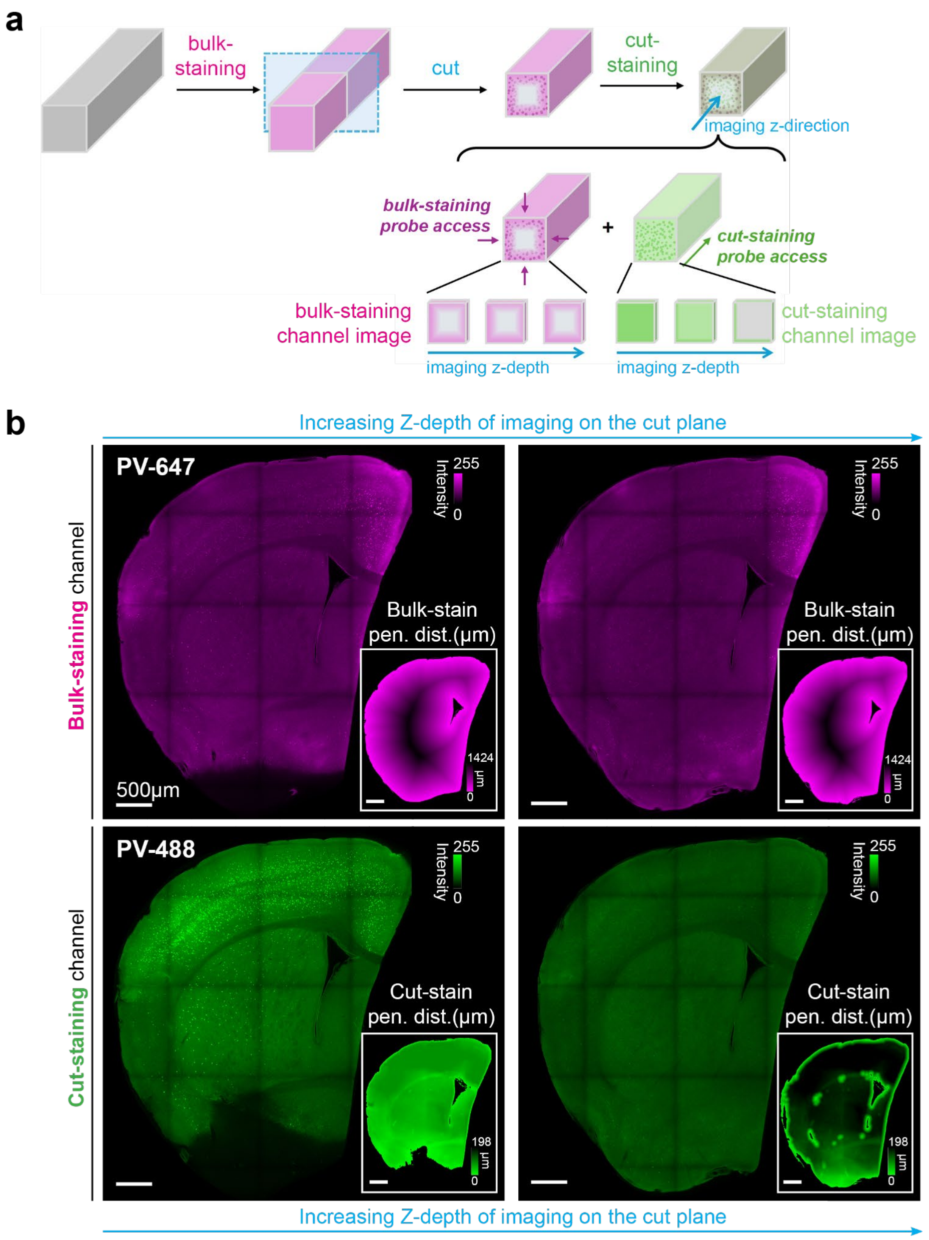

Supplementary Figure 4. Evaluating deep tissue staining methods via bulk and cut staining. a Benchmarking Experiment Design and Principle Illustration. A tissue block (grey) is first stained with the benchmarked method for a marker of choice (magenta, bulk staining). The stained tissue is then cut and restained under conventional staining conditions (e.g., PBS with 0.1% Tween 20) for the identical marker with a fluorophore with non-overlapping spectrum as the previous step (green, cut staining). The double-stained tissue is then imaged on the cut plane, where the comparison between the bulk-staining channel images (benchmarked method) and the cut-staining channel images (expected staining pattern) shall demonstrate the efficacy of the benchmarked staining method. In the illustration, the benchmarked method (magenta) shows a rimming pattern with failure to reproduce the pattern in cut-staining (green), indicating limited probe penetration. **b** Typical imaging results from a sample in the benchmarking experiment. The bulk-staining was conducted before tissue cutting, so the bulk-staining intensities (upper row) and their corresponding calculated penetration lengths (insets of upper row) are relatively invariant at the same *z*-depth of imaging (arranged in columns). Conversely, cut-staining was performed after tissue cutting, and antibodies could penetrate through the cut surface, which corresponds to the imaging plane, displayed in the *xy*-plane here (lower row). The penetration distance was irregular and inhomogeneous due to minor tissue curvatures and irregular tissue texture, and areas of lower penetration distances due to vasculatures. These factors were taken into account during the quantification of cut-staining penetration depth (insets of lower row).

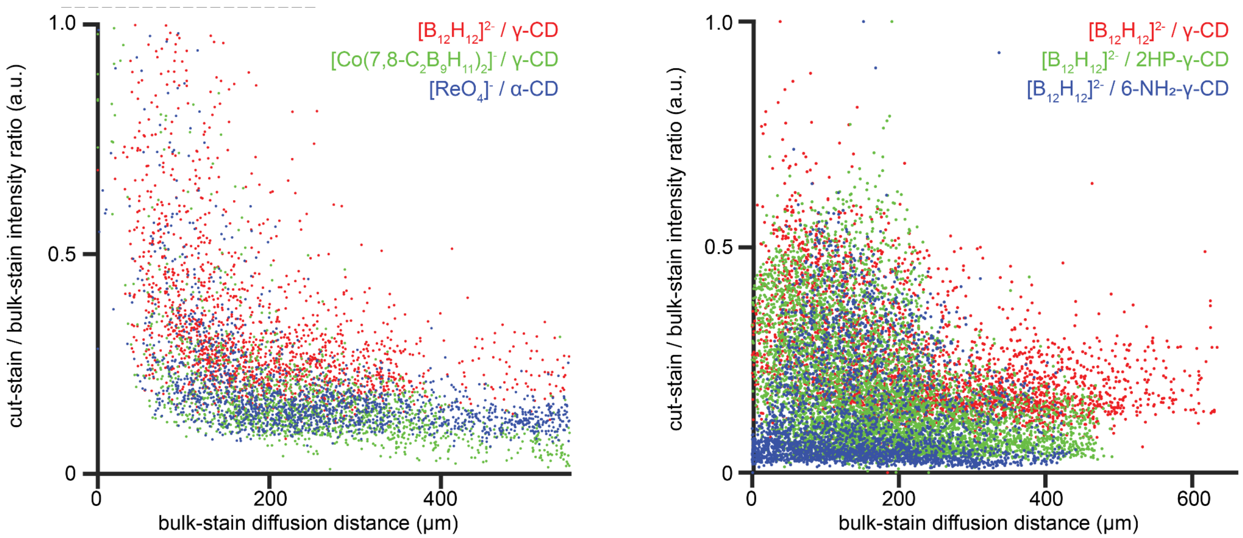

Supplementary Figure 5. Evaluation of different weakly coordinating superchaotropes (left panel) and γ-cyclodextrin derivatives (right panel) both at 0.1M concentration using the benchmarking experimental pipeline as described previously [12,13].

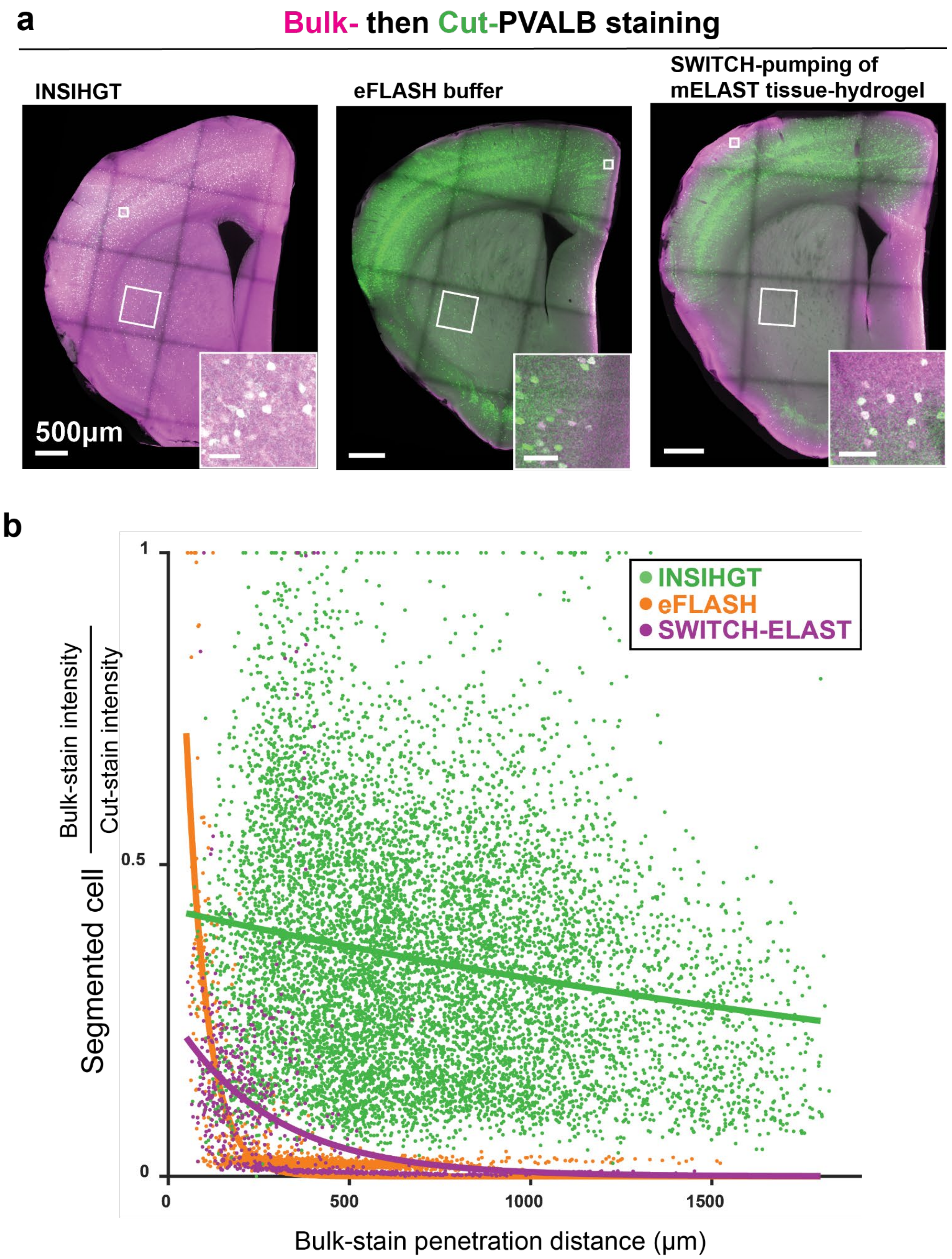

Supplementary Figure 6. Performance Comparison of INSIHGT with eFLASH and SWITCH. a Comparison of INSIHGT against binding kinetics modulatory buffers in eFLASH and SWITCH-pumping of mELAST tissue hydrogel buffer. Panel of INSIHGT is the same as **Fig 2b**. **b** Quantification of bulk:cut-staining signal ratio against penetration distance for segmented cells in the same procedures described in **Fig. 2b**.

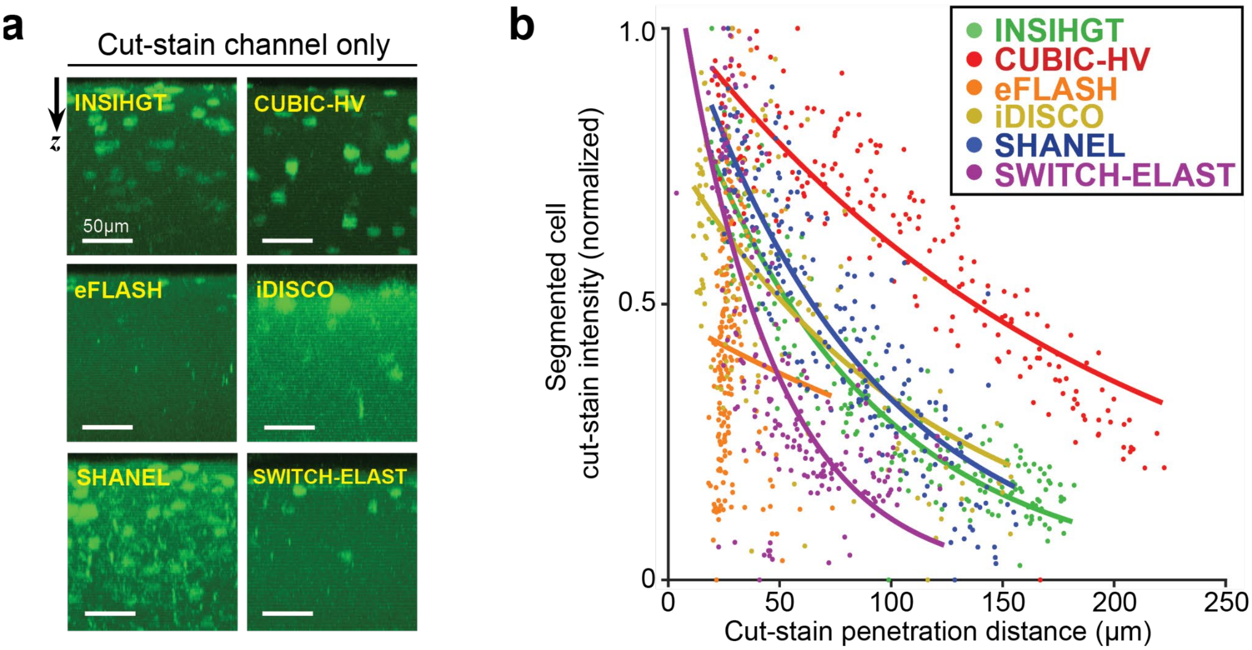

Supplementary Figure 7. Analysis of staining efficacy of various methods. a *x-z* projection of larger white boxed areas from **Figure 2b and S6a** at deep bulk-staining penetration depths, with only the cut-staining channels (without the use of any deep immunostaining methods) shown. The deeper the penetration, the more permeabilized the tissue. **b** Quantitative comparison of tissue permeabilization by correlating cut-staining penetration depth and cut-staining intensity.

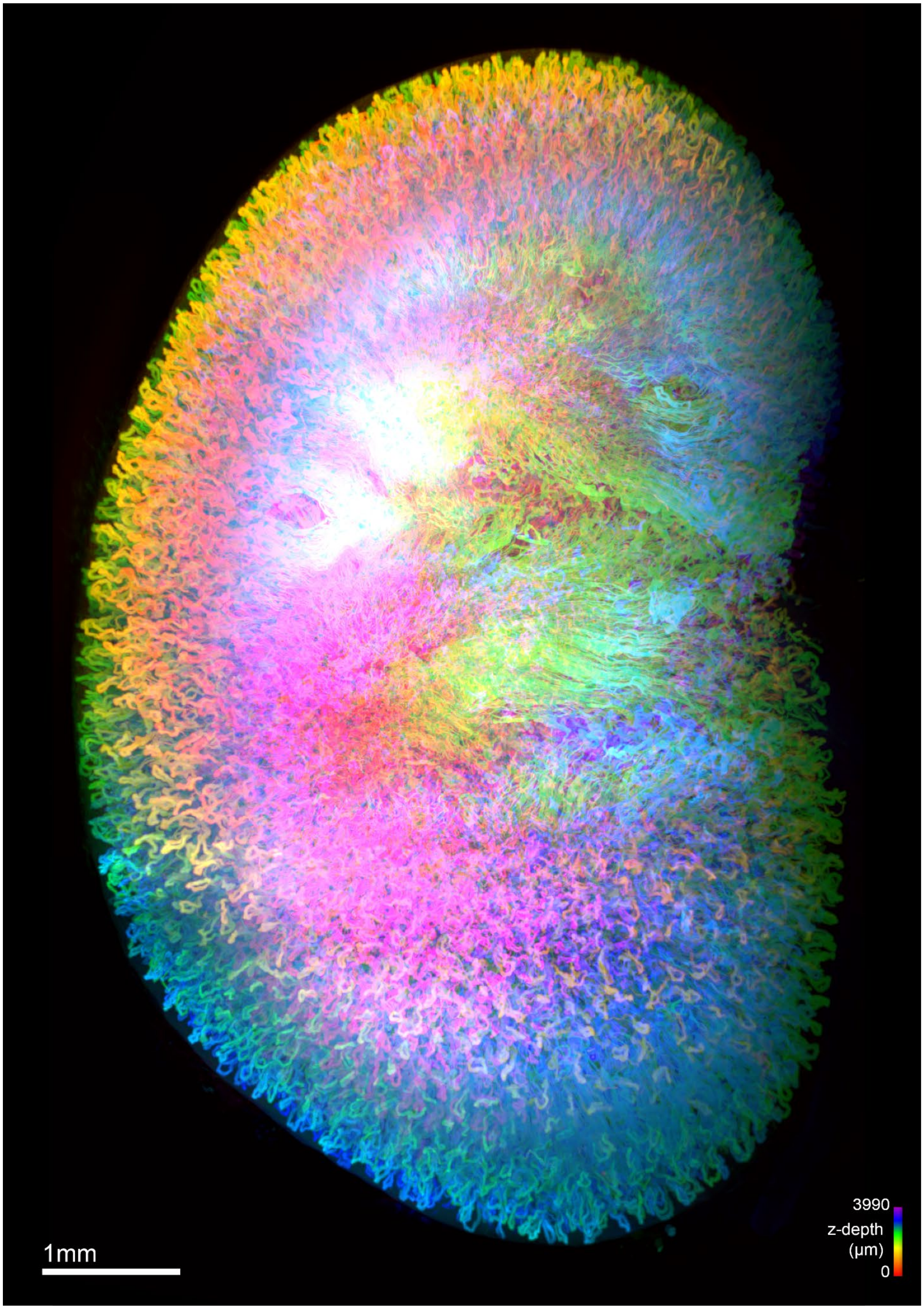

Supplementary Figure 8. Whole mouse kidney stained with Lycopersicon esculentum (Tomato) Lectin. 3D rendering, colour-coding: z-depth.

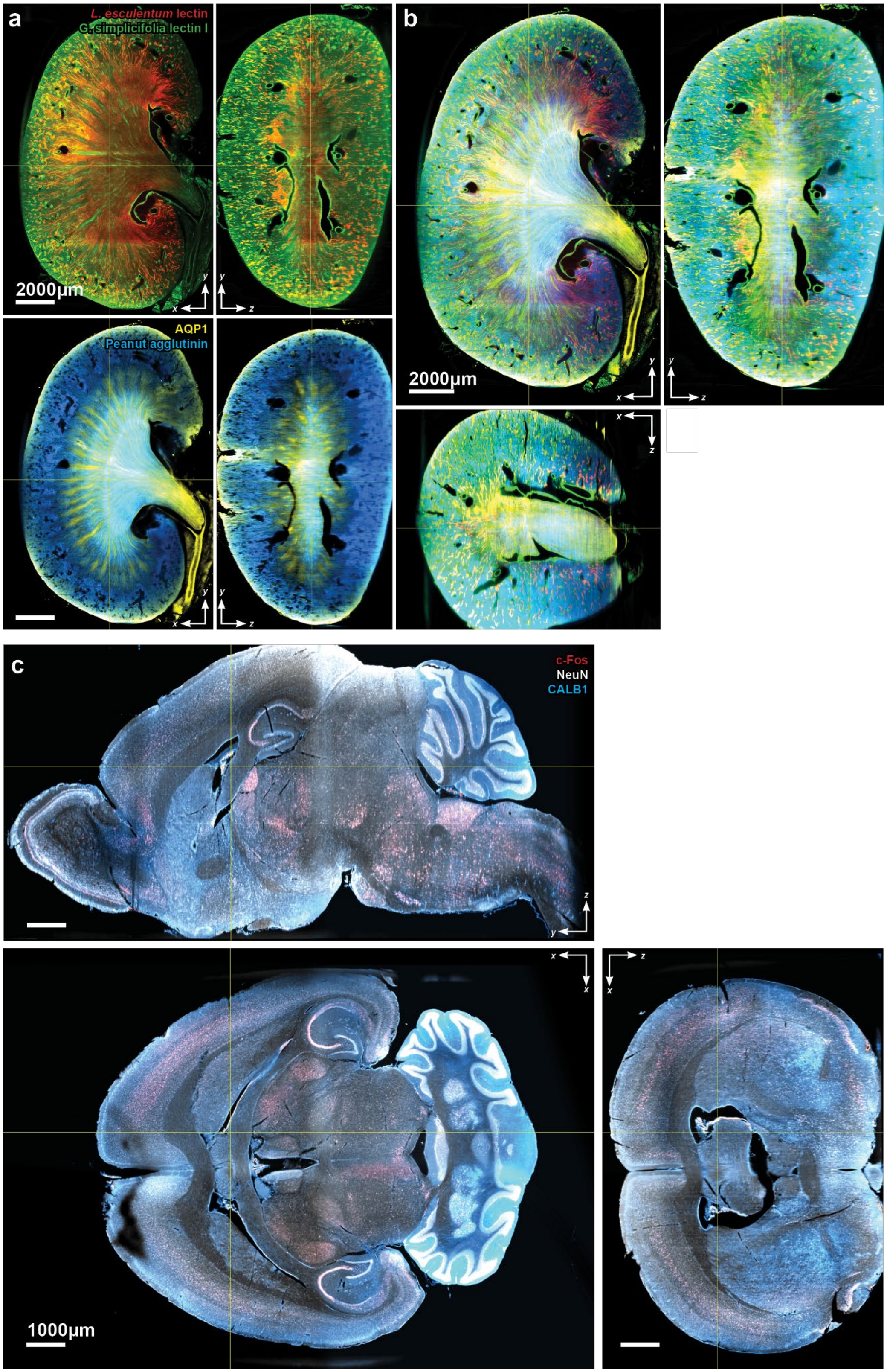

Supplementary Figure 9. Orthogonal views of densely labelled samples in Fig 3. a-b Orthogonal views of the 4-plex sample in **Fig 3c-d**. **c** Orthogonal views of the 3-plex 36-month-old (biological age) mouse brain sample in **Fig 3e-i**.

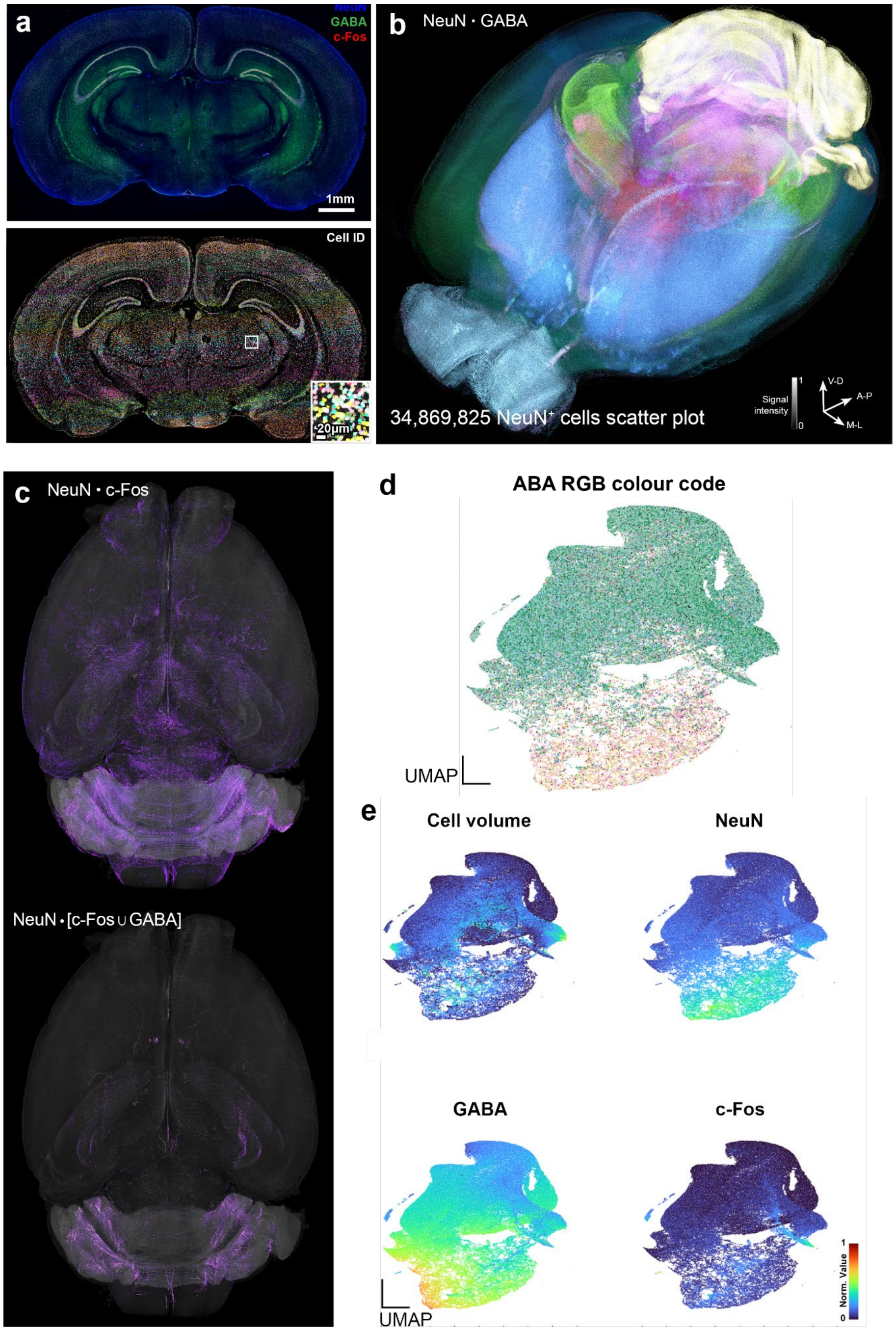

Supplementary Figure 10. INSIHGT enables whole brain structural molecular functional quantification. a Whole mouse brain multiplexed mapping of over 34 million neurons by molecular (NeuN), neurotransmitter type (GABA), and functional (c-Fos) mapping. An example coronal slice and its corresponding 3D segmentation results are shown in the upper and lower panels, respectively. Inset in the lower panel shows the cell masks in the white boxed area. **b** Rendered view of all segmented cells, color-coded according to brain regions with intensity modulated by a combination of marker levels. The RGB color codes for the brain regions are based on that of the Allen Brain Atlas CCFv3. **c** Whole-brain active neurons and GABAergic neurons inferred by double positivity in c-Fos and NeuN (upper panel) and triple positivity in c-Fos, NeuN and GABA (lower panel). **d** UMAP embedding of 0.35M (1%) segmented NeuN+ cells in **b** with spatial regional classification. UMAP embedding was based on cell volume, NeuN and c-Fos expression levels and GABA levels detected with INSIHGT. These normalized values were color-coded in **e**.

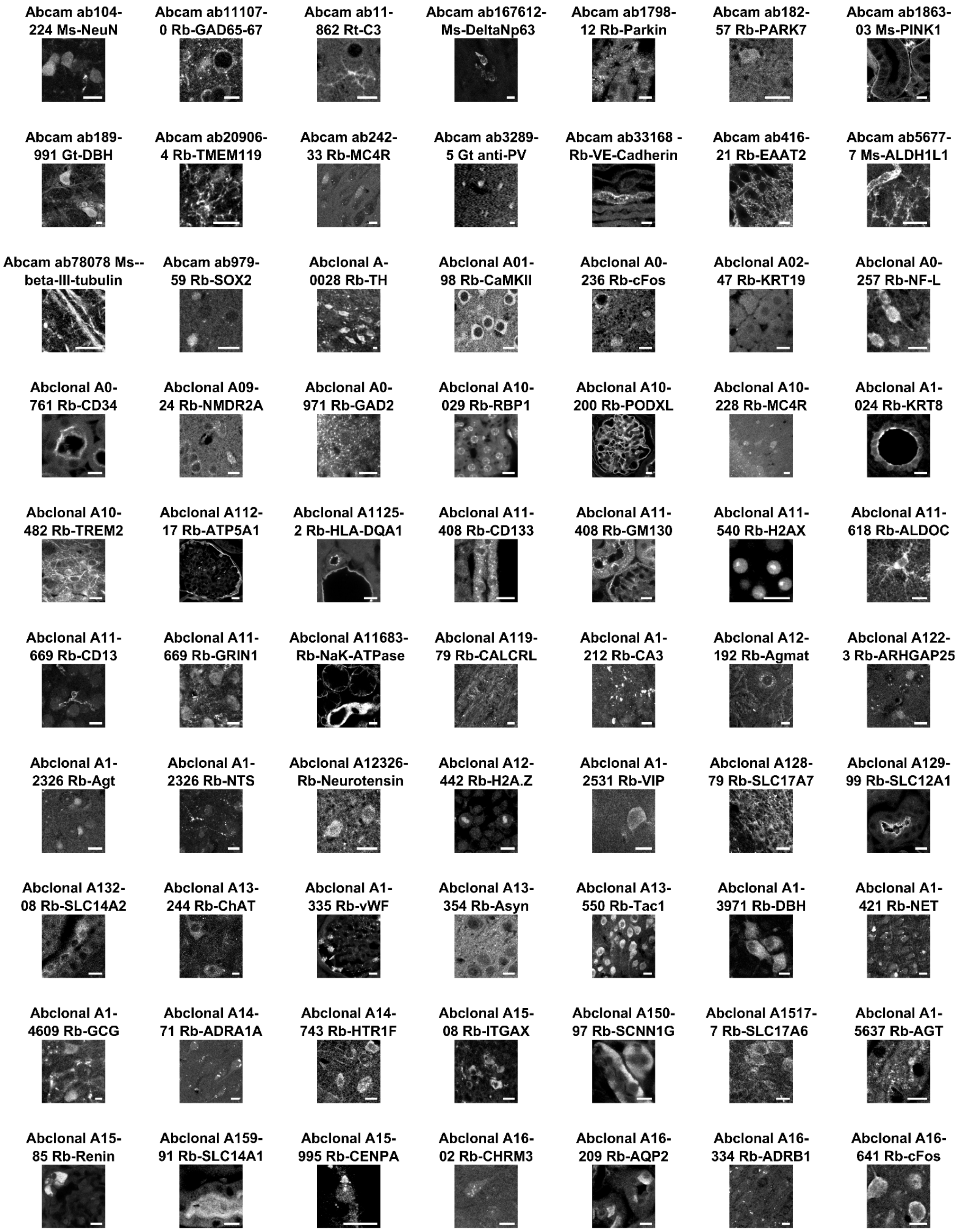

Supplementary Figure 11. Representative images from INSIHGT-compatible antibodies, Part 1. Scale bars are set to 10 µm.

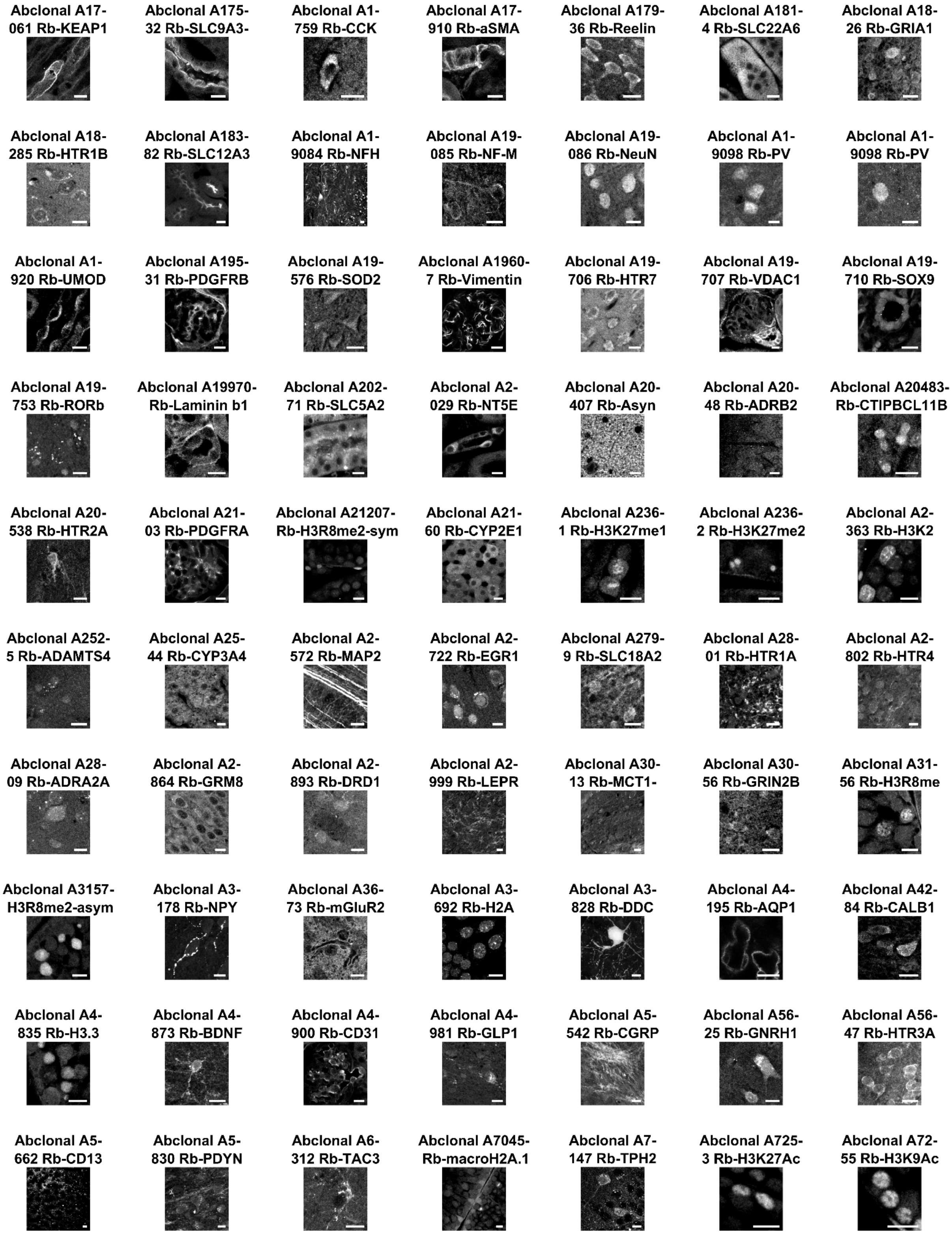

Supplementary Figure 12. Representative images from INSIHGT-compatible antibodies, Part 2. Scale bars are set to 10 µm.

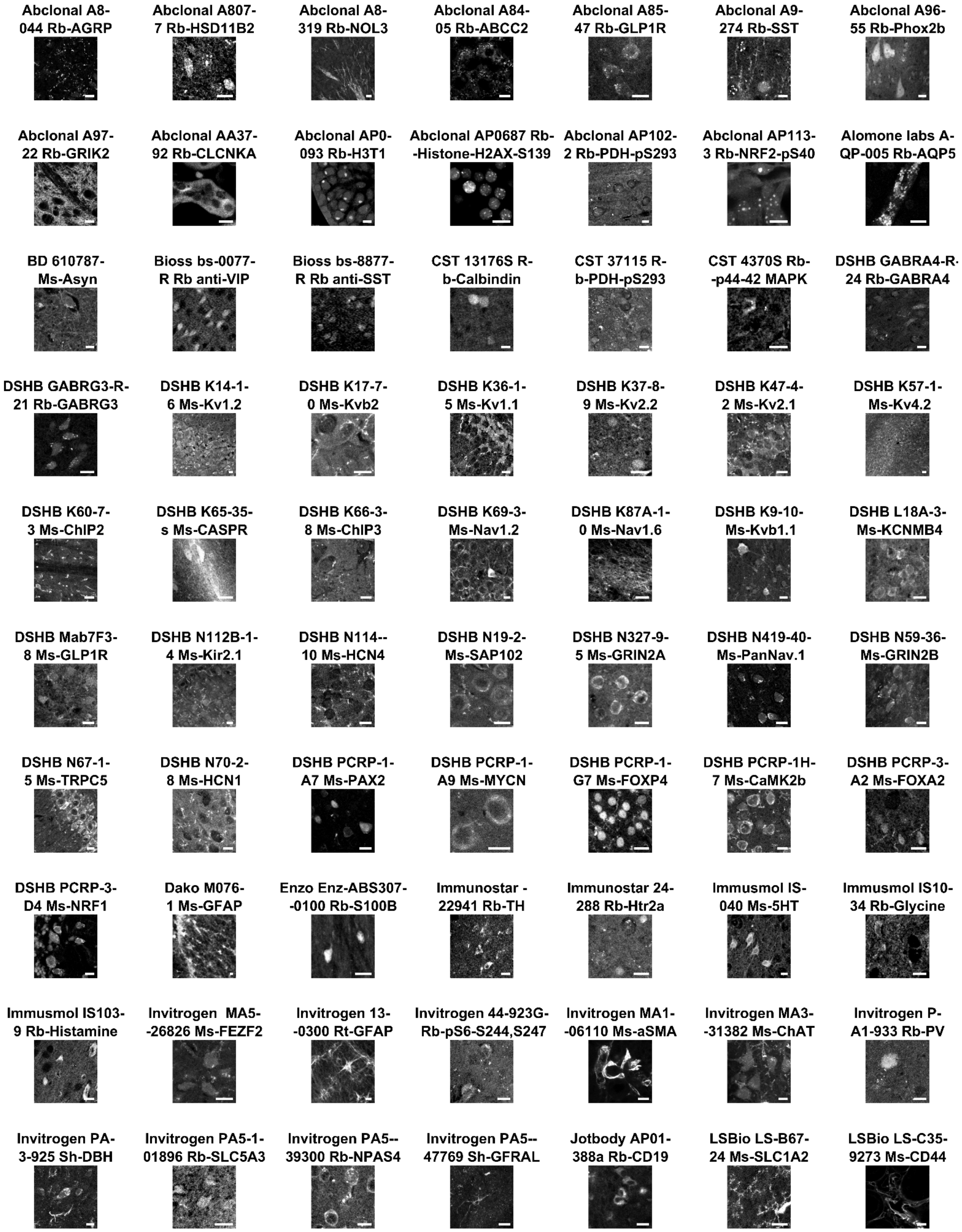

Supplementary Figure 13. Representative images from INSIHGT-compatible antibodies, Part 3. Scale bars are set to 10 µm.

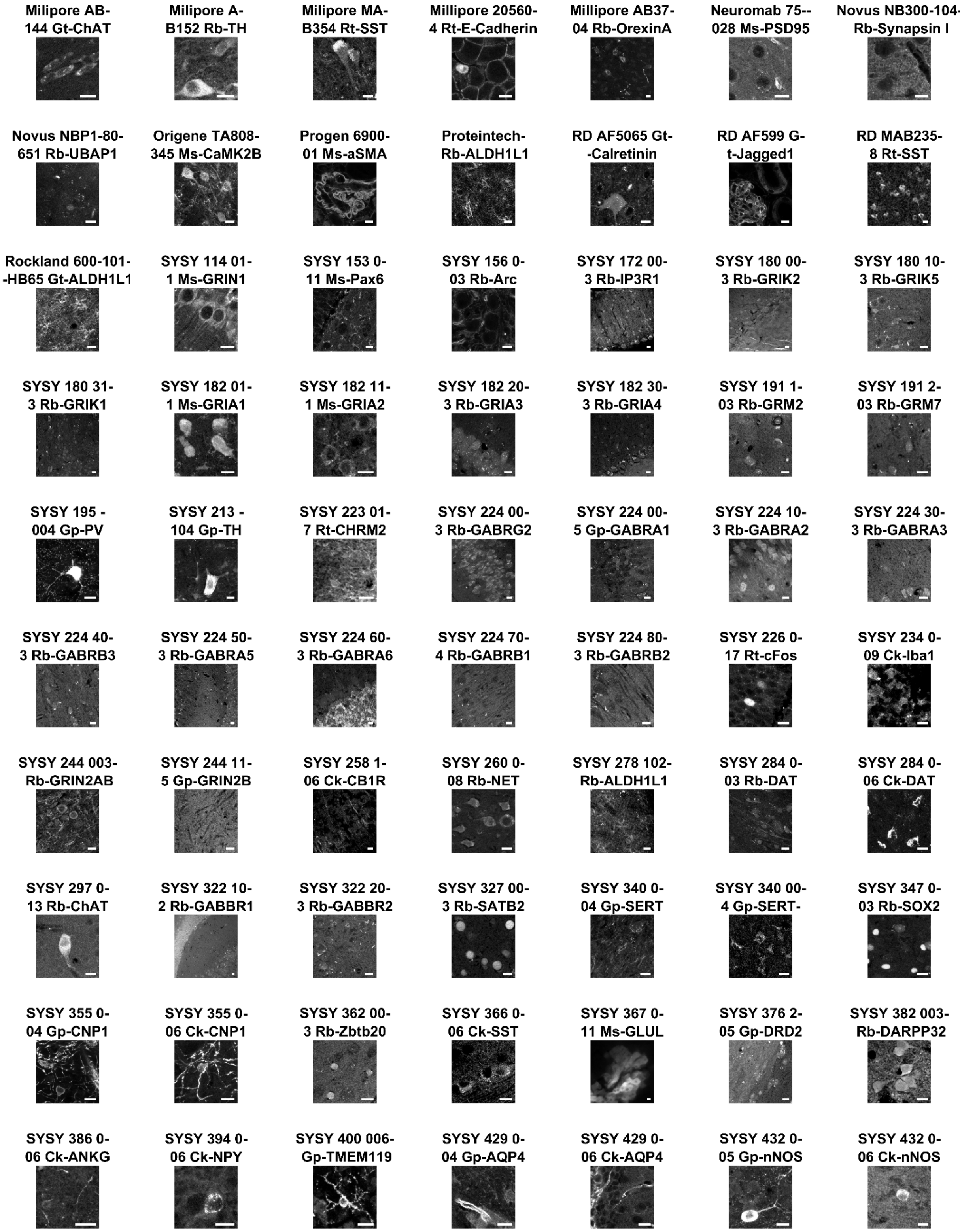

Supplementary Figure 14. Representative images from INSIHGT-compatible antibodies, Part 4. Scale bars are set to 10 μm.

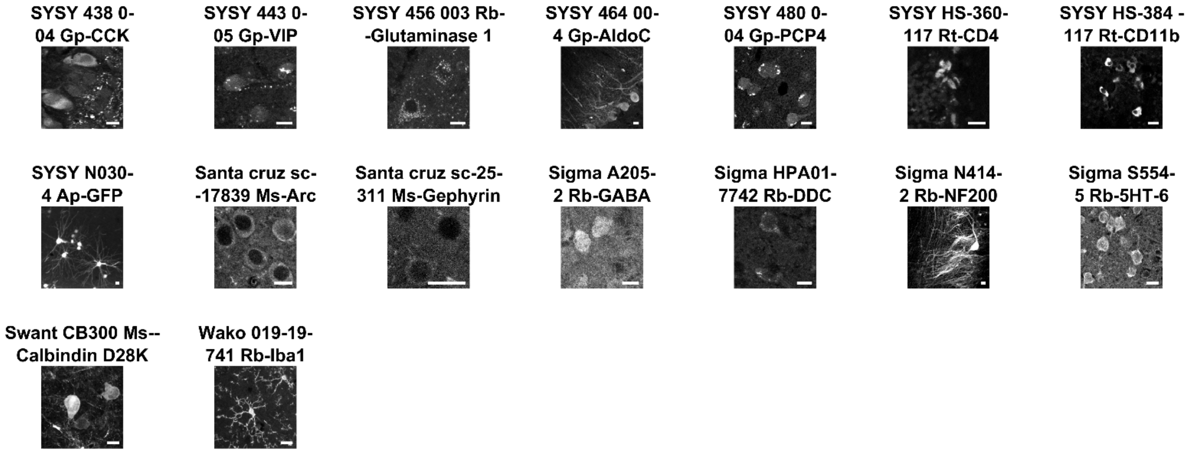

Supplementary Figure 15. Representative images from INSIHGT-compatible antibodies, Part 5. Scale bars are set to 10 µm.

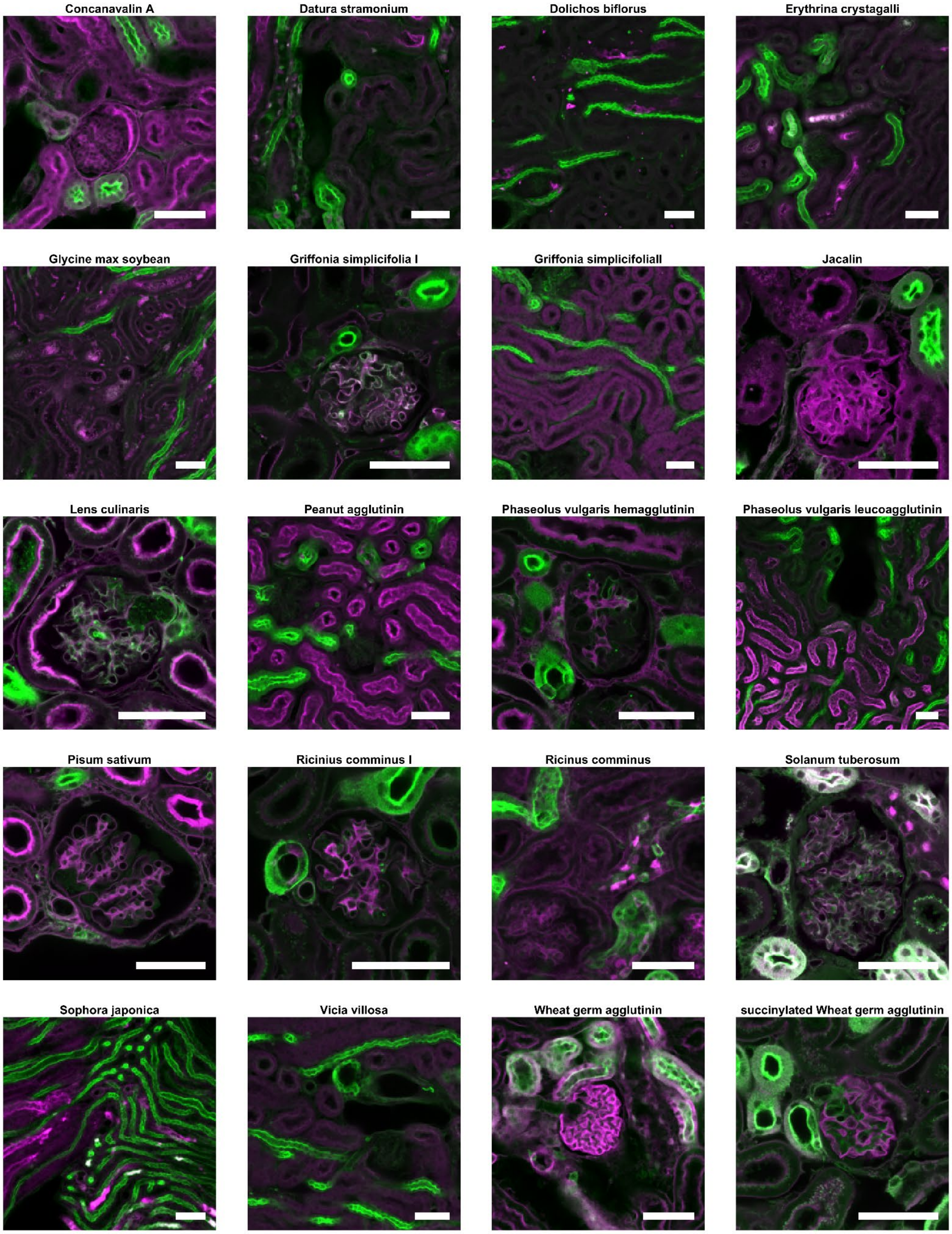

Supplementary Figure 16. Representative images of lectins histochemistry compatible with INSIHGT performed on mouse kidney tissue. Shown in magenta are the titled lectin's signals. The signals from *Lycopersicon esculentum* lectin are shown in green for orientation. Scale bars are set to 50 μm.

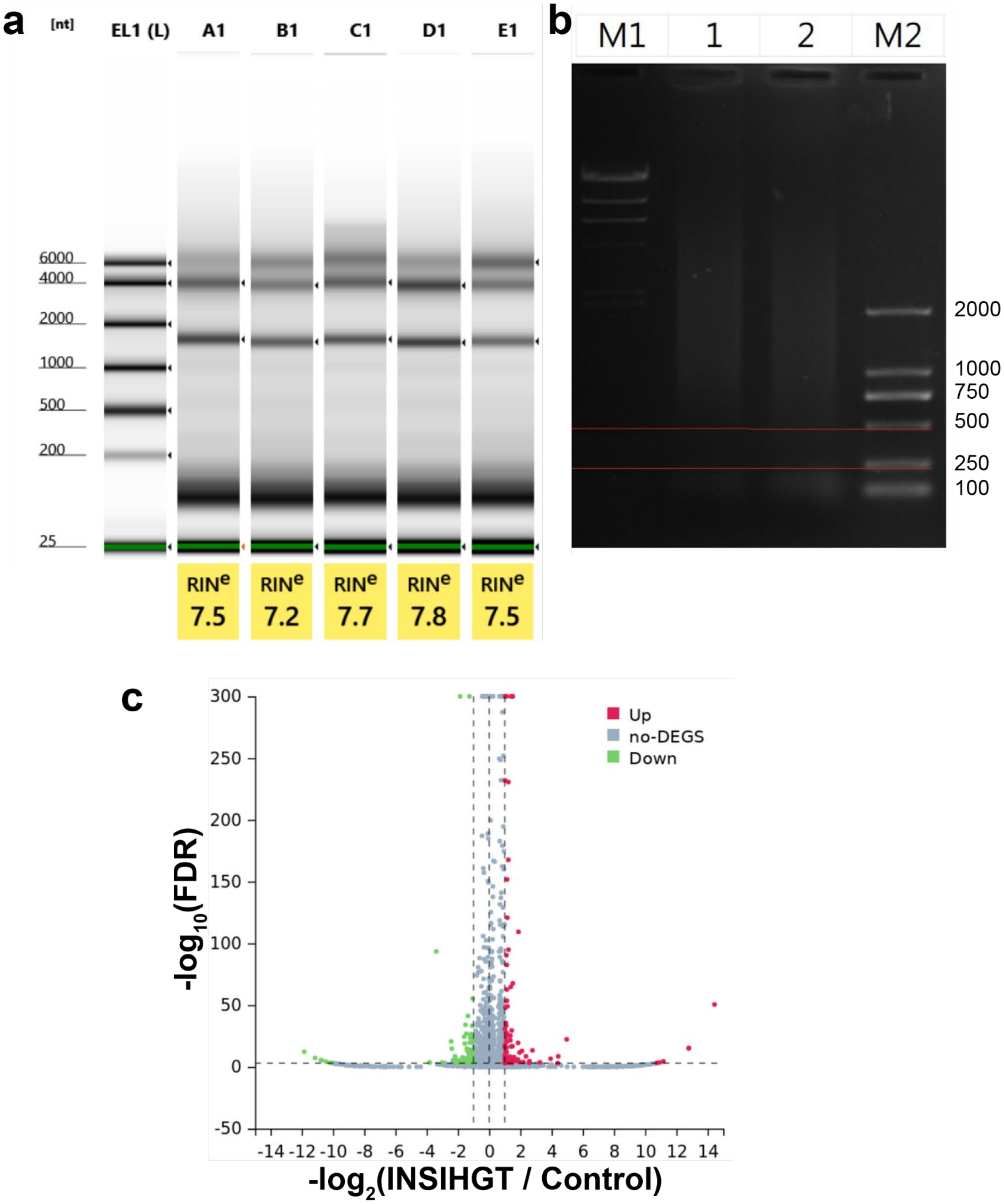

Supplementary Figure 17. RNA integrity analysis and whole genome DNA extraction electrophoretogram reveals non-destructiveness of INSIHGT. a RNA integrity number (RIN) analysis of INSIHGT-retrieved tissues. The wells contain samples that are (A1) control, (B1) with delipidation, (C1) with INSIHGT solution A treatment, (D1) with INSIHGT solution B treatment, (E1) with BABB clearing. **b** Whole genome DNA extraction electrophoretogram. (M1) λ-Hind III digest DNA (Takara), (M2) D2000 (Tiangen). (1) Control, (2) INSIHGT-treated sample following our described full pipeline with 3 days of INSIHGT solution A incubation. DNA total mass for control (14.588 μg) and INSIHGT-treated sample (10.12 μg), and their DNA length (DNAfragment ≥ 500bp) are comparable. **c** Volcano plot of differentially expressed genes between control (non-INSIHGT-treated) and INSIHGT-treated samples. The INSIHGT-treated sample was performed with 3 days of incubation in INSIHGT buffer A, 1 day of incubation in INSIHGT buffer B, cleared with BABB, and retrieved with PBS washing for RNA extraction and sequencing.

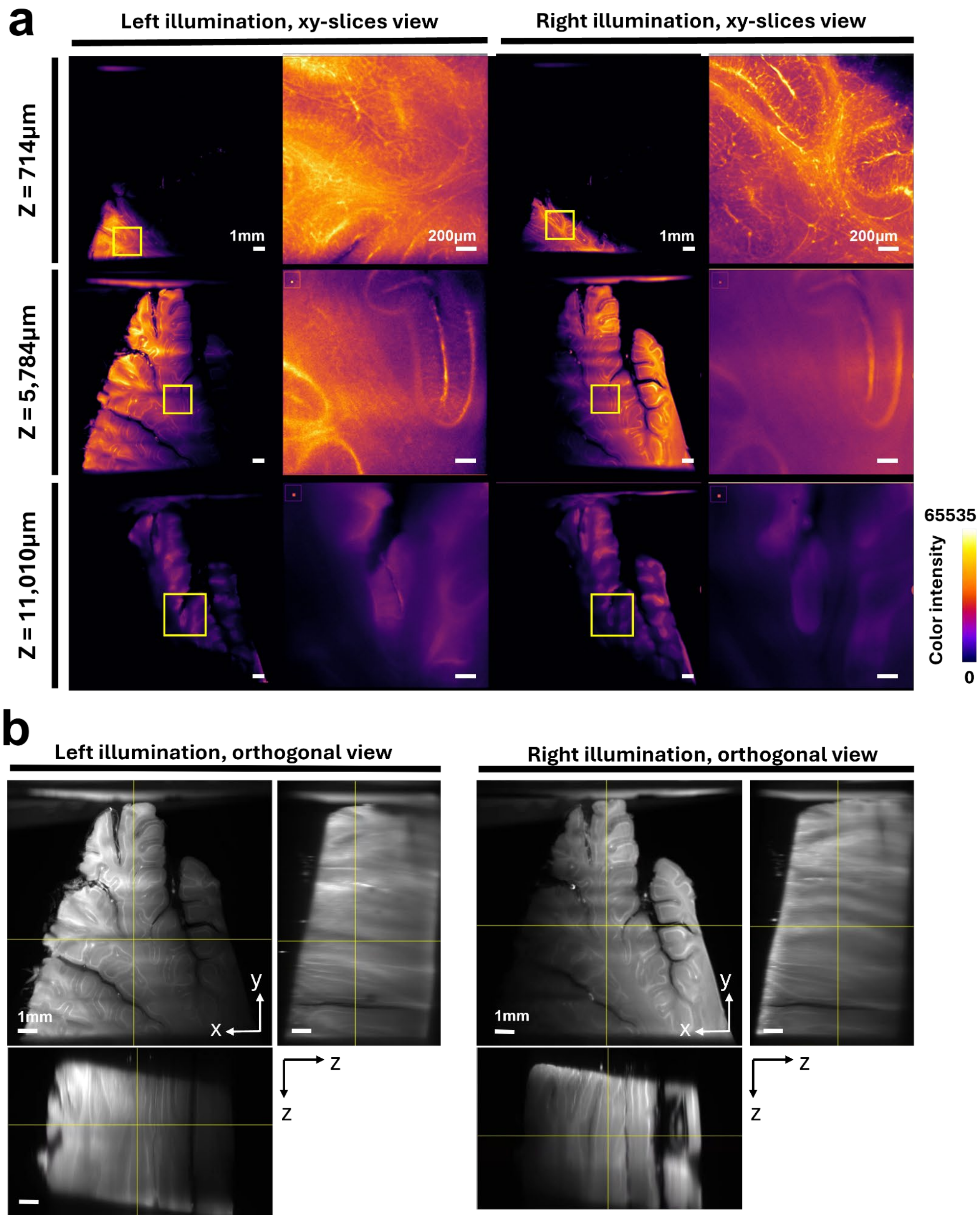

Supplementary Figure 18. Light-sheet microscopy-based assessment of macromolecular probe penetration and its limitations. a Attempted MesoSPIM imaging of the same human cerebellum sample as shown in **Fig. 5d - f.** Due to the strong light scattering and absorbance in such a large, pigmented sample, excitation light penetration is limited, as evident by the differences in left versus right laser illumination. The image quality also deteriorates along the z-direction as the emission light must travel through the thick sample to reach the camera. Light-sheet microscopy is hence suboptimal in the evaluation of probe penetration for large human tissues. Scale bars are 1mm in the overview and 200µm for the enlarged views of the yellow boxes. **b** Orthogonal views of the same sample under left and right illumination, illustrating the deterioration of image quality along the z-direction. Scale bars, 1mm.

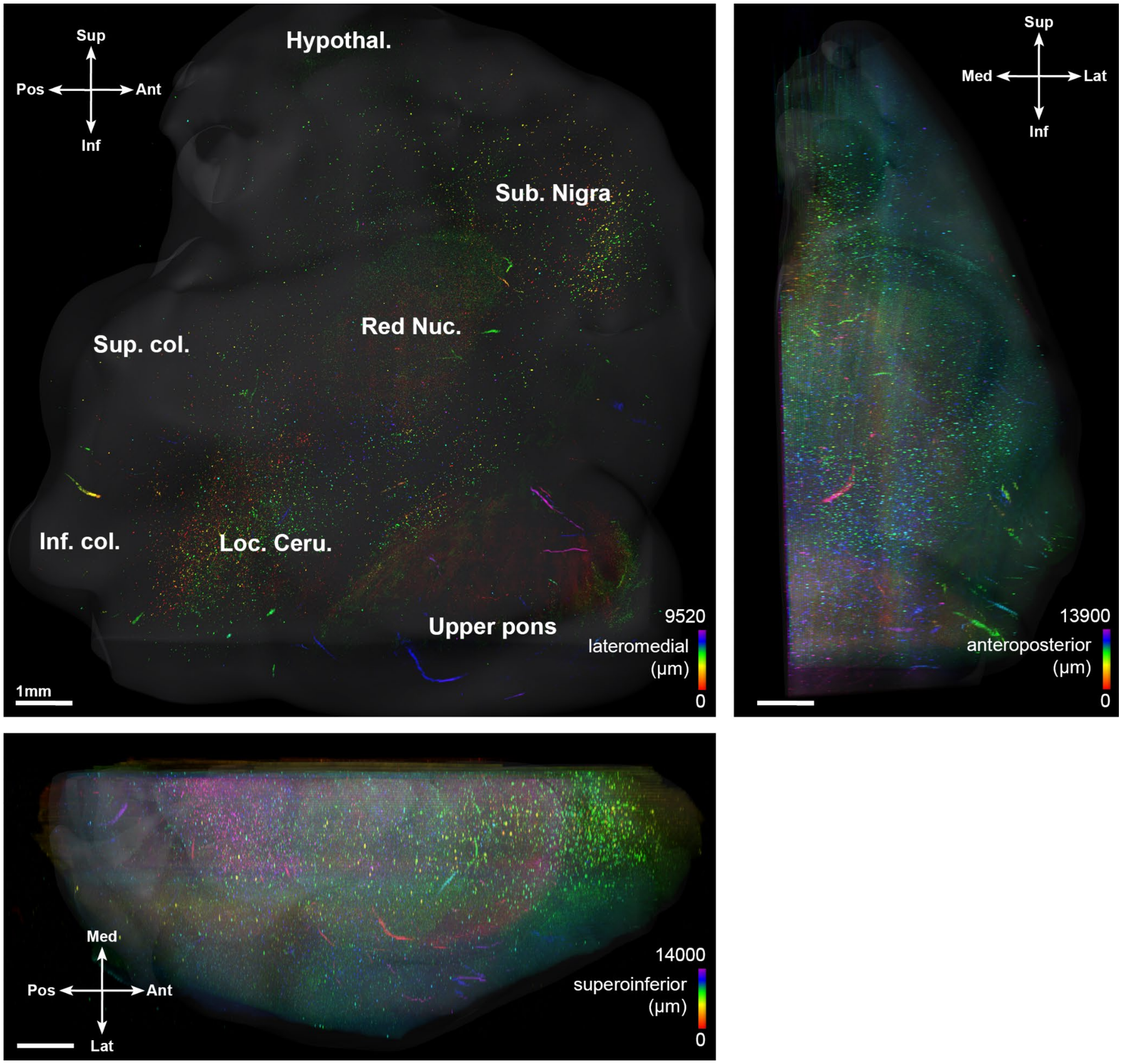

Supplementary Figure 19. Overview of the human brainstem stained for alpha-synuclein phosphorylated at serine 129 shown in Fig. 5g-i. The INSIHGT immunostaining signal was color-coded according to the dimension going in and out of the paper plane. The surface rendering provides a rough view of the tissue contour. Hypothal.: hypothalamus, Loc. Ceru.: locus ceruleus, Inf. col.: inferior colliculus, Sub. Nigra: substantia nigra, Sup. col., superior colliculus.

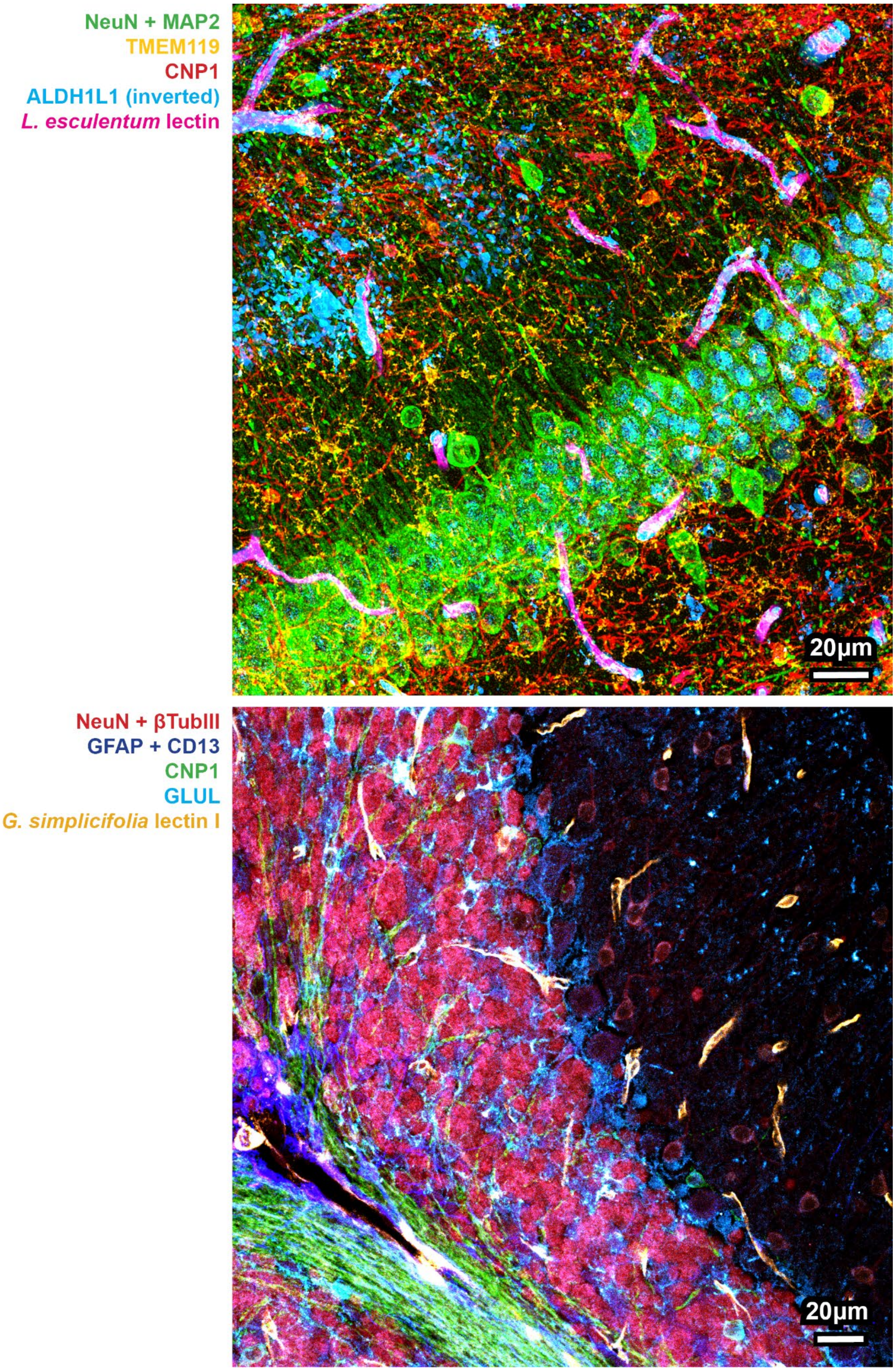

Supplementary Figure 20. Multiplexing with INSIHGT with high concentrations of antibodies. Upper image: mouse hippocampus stained with 6 protein probes, lower image: mouse cerebellum stained with 7 protein probes. The signal of ALDH1L1 was inverted due to the ubiquitous homogeneous staining of the original signal.

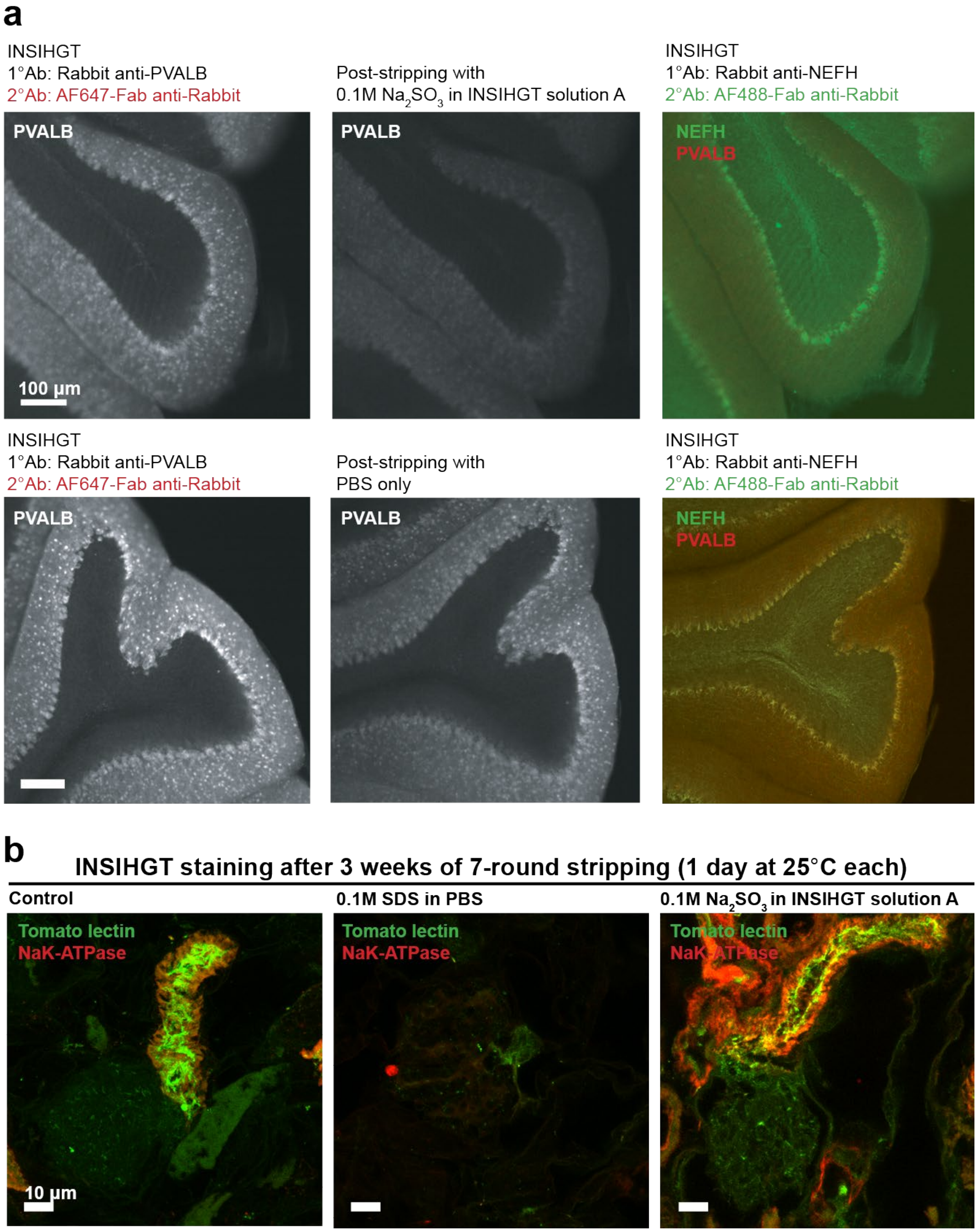

Supplementary Figure 21. Antibody stripping and re-probing. a $[B_{12}H_{12}]^{2-}$ in 1x PBSN in the presence of 0.1M Na_2SO_3 effectively strips away the rabbit antibody staining from INSIHGT-stained slices (upper row), while PBS alone would not lead to stripping of antibodies (lower row). After stripping, a second round of INSIHGT-based rabbit anti-NEFH staining was applied to demonstrate the striped rabbit IgGs within the tissue. **b** 4% PFA-fixed mouse kidney undergone 7 weeks of stripping with SDS versus Na_2SO_3. The total incubation time was 3 weeks. The samples were then re-stained with INSIHGT protocol using tomato lectin (in green) and rabbit anti-NaK-ATPase antibodies (in red).

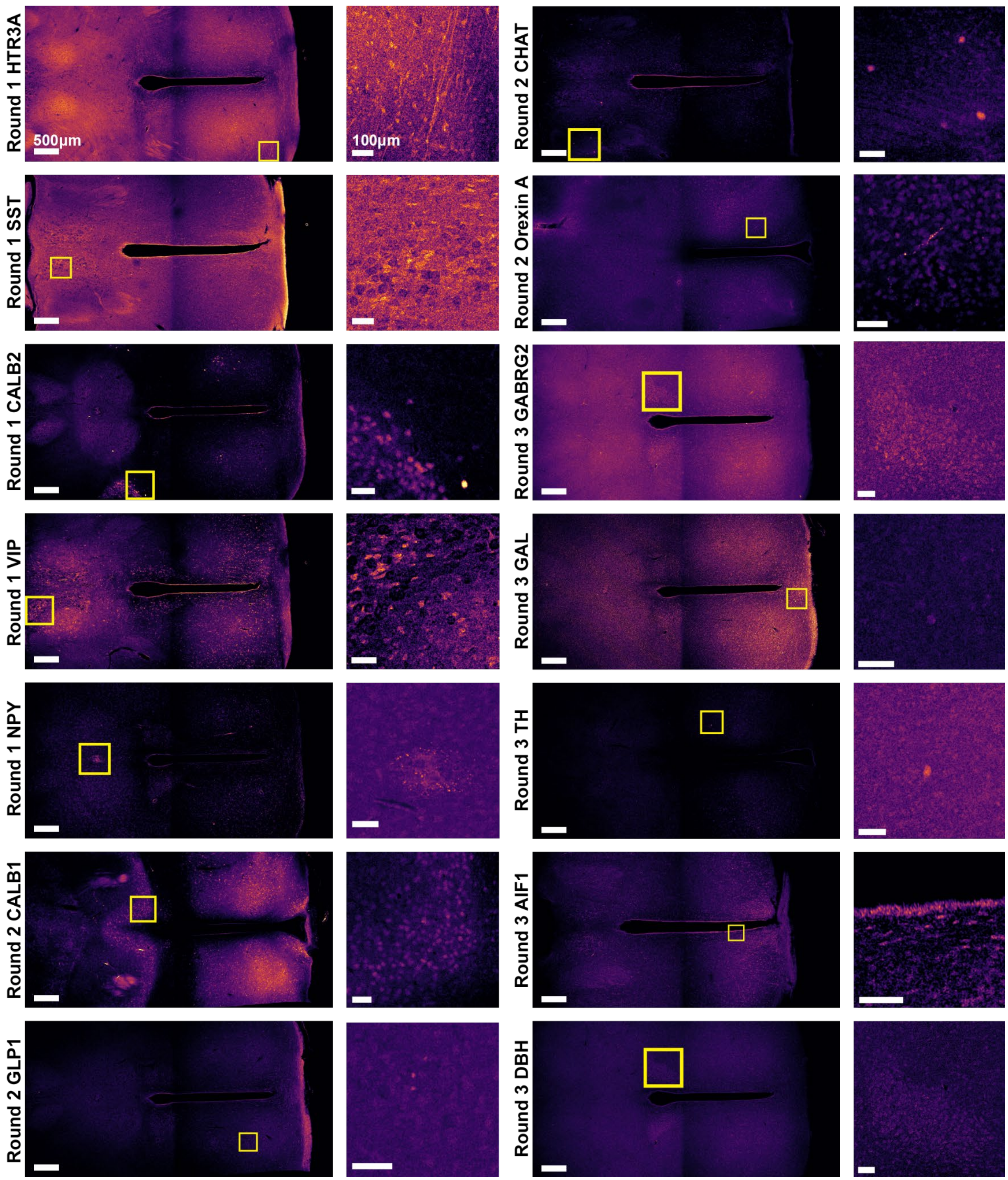

Supplementary Figure 22. Raw aligned images of multi-round imaging of the mouse hypothalamus in Fig. 5, Part 1. A single z-slice was excerpted from each image volume for clarity, and enlarged views from yellow-boxed regions were shown adjacent to the tile overview images. Proteins are named based on their gene names or explicitly stated otherwise (for Orexin-A and glucagon-like peptide, GLP1). Scale bars for overview images: 500μm, enlarged view images: 100μm.

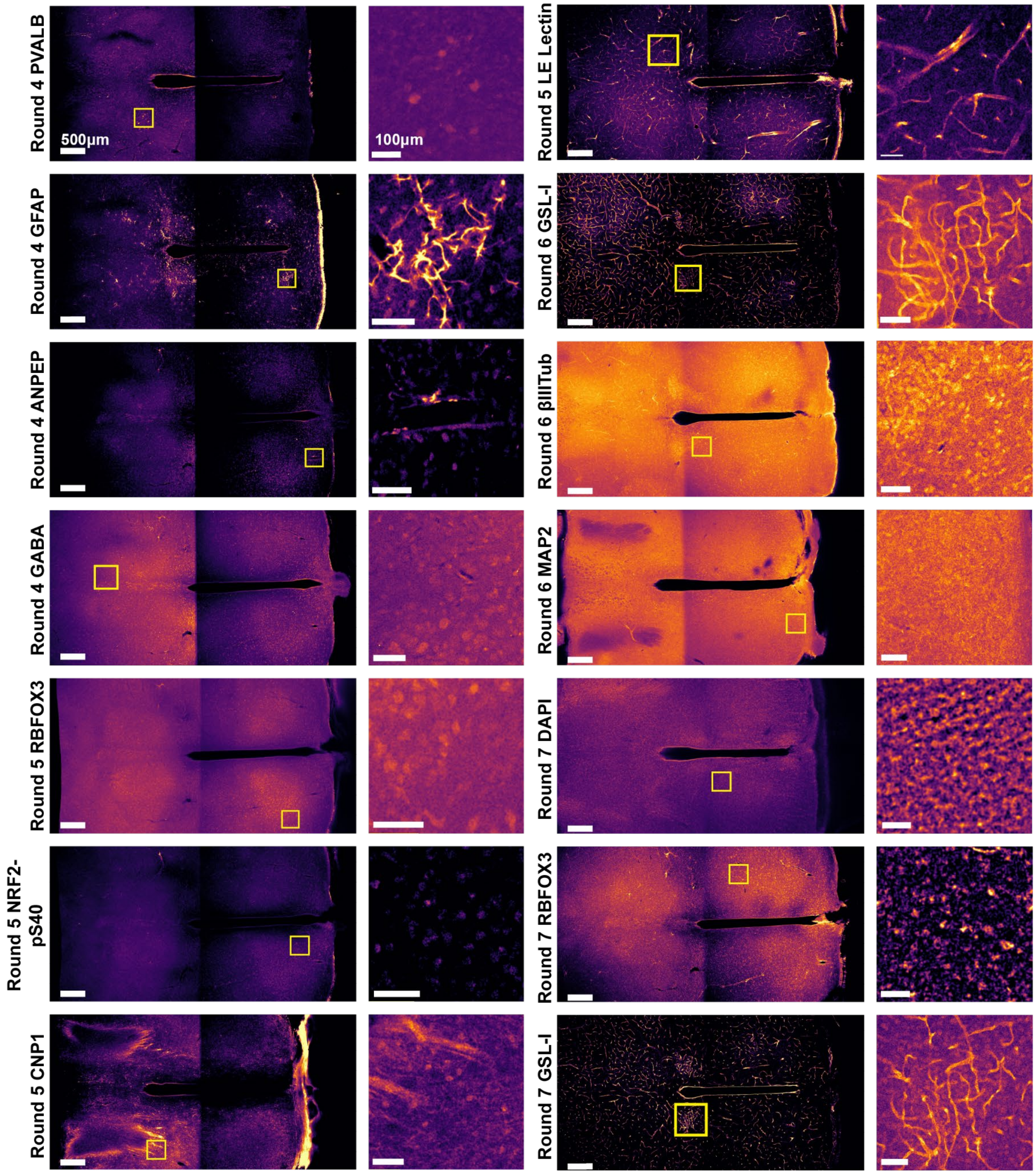

Supplementary Figure 23. Raw aligned images of multi-round imaging of the mouse hypothalamus in Fig. 5, Part 2. A single z-slice was excerpted from each image volume for clarity, and enlarged views from yellow-boxed regions were shown adjacent to the tile overview images. Proteins are named based on their gene names, except for GABA (γ-aminobutyric acid), NRF2-pS40 (NRF2 phosphorylated at serine-40 residue), LE lectin (*Lycopersicon esculentum* Lectin), GSL-I *(Griffonia Simplicifolia* Lectin I), βIIITub (Tubulin beta-3 chain), DAPI (4′,6-diamidino-2-phenylindole). Scale bars for overview images: 500µm, enlarged view images: 100µm.

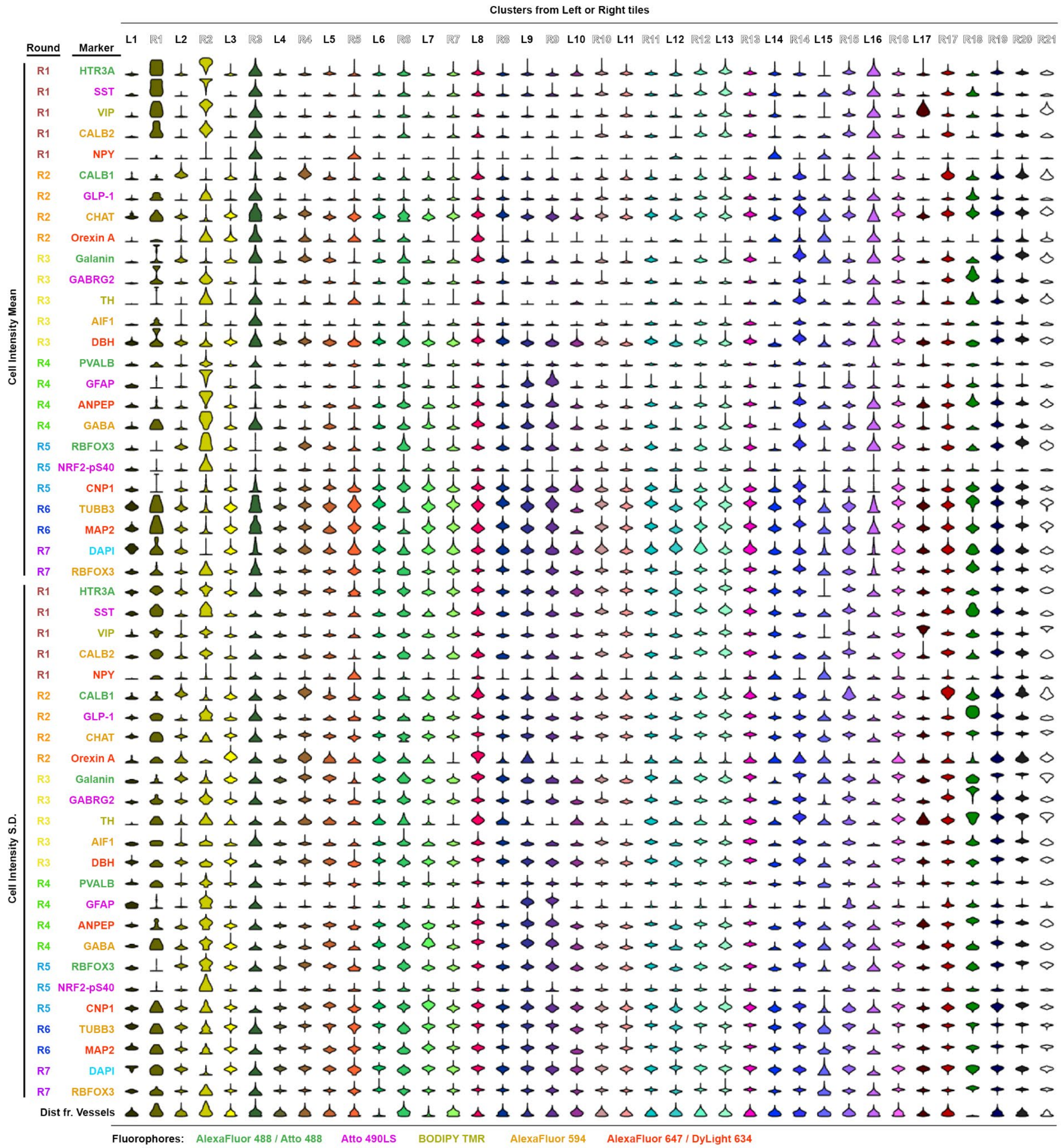

Supplementary Figure 24. Violin Plots of Each Marker. The plot represent immunostaining mean intensities and their standard deviations (SDs), separately, and the distance from vessels for each cluster obtained by nested UMAP analysis in **Figure 6**. The text is color-coded based on the fluorophore used in the imaging experiment.

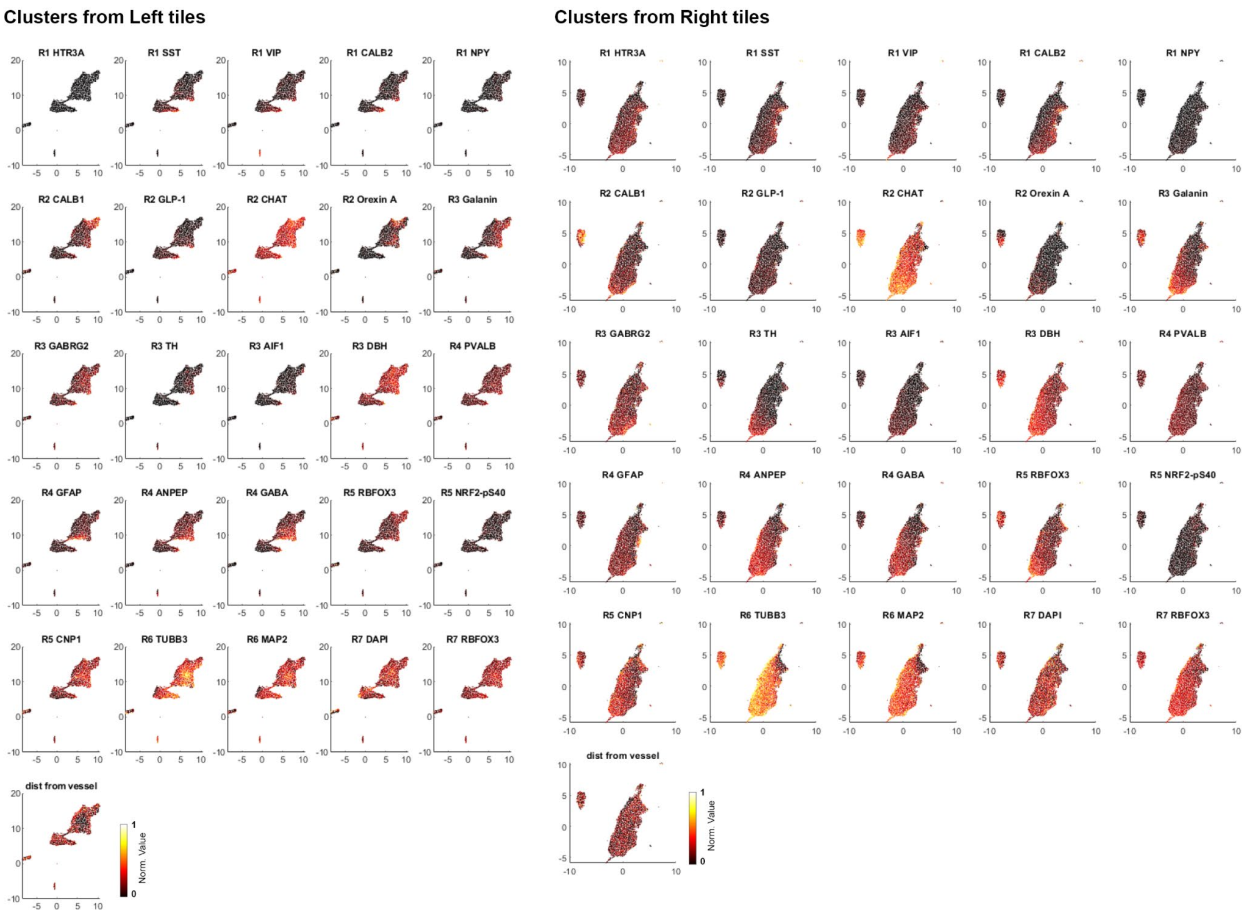

Supplementary Figure 25. The mean immunostaining intensities of each marker in a 2D-embedded UMAP space.

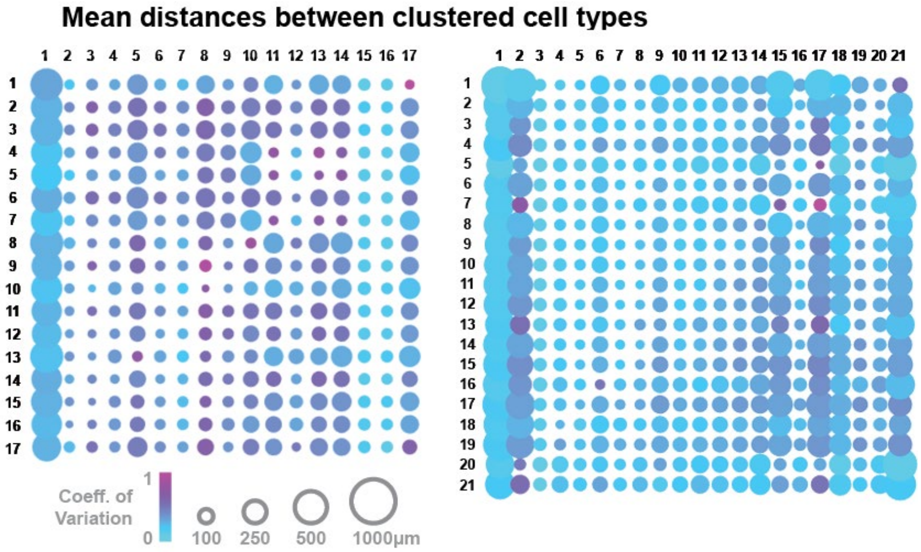

Supplementary Figure 26. Mean nearest distance matrix between each cell type from each cluster. The size of the dot plot represents the coefficient of variation.

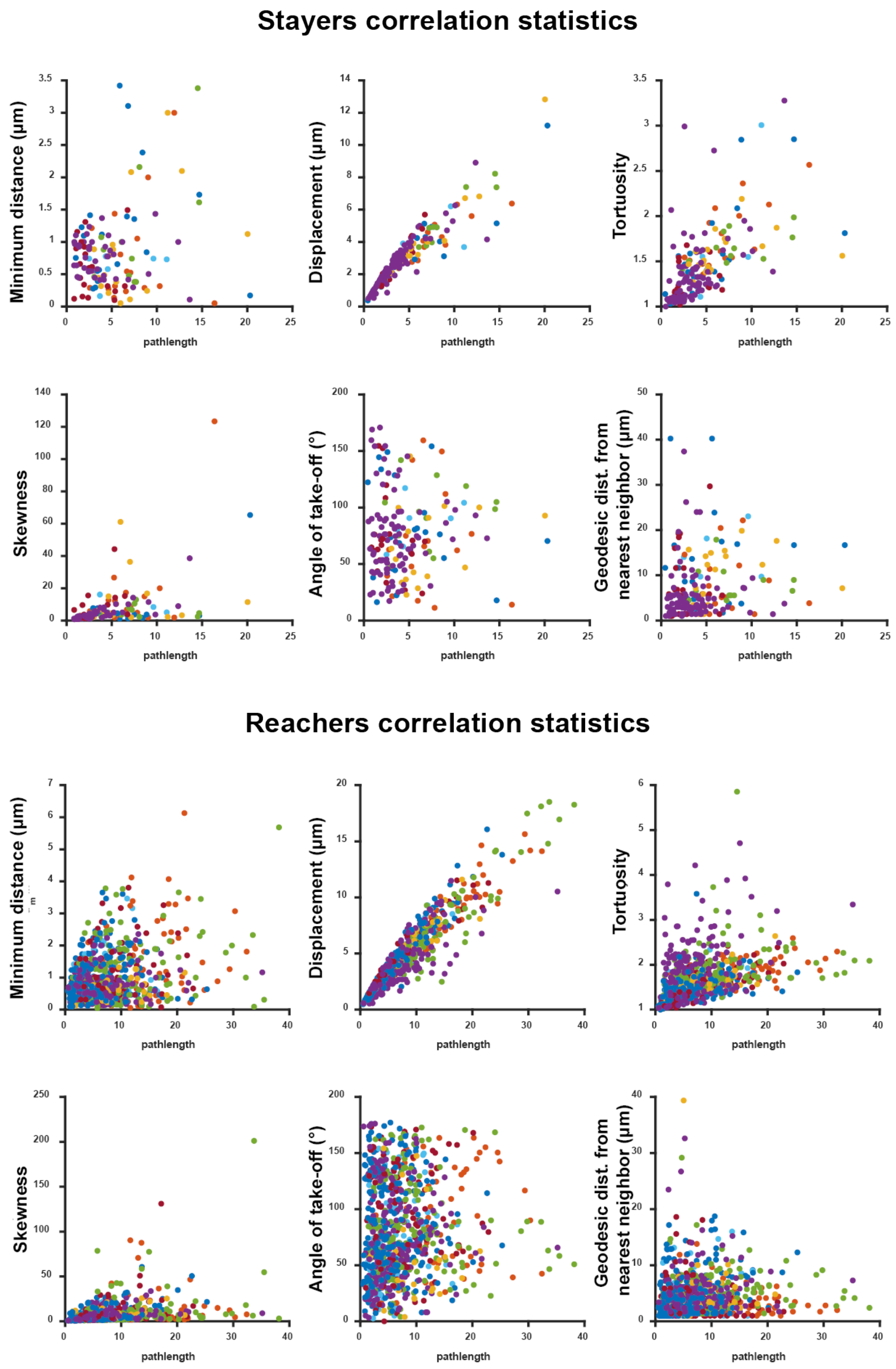

Supplementary Figure 27. Correlation statistics of physical parameters obtained from 3D images of mouse kidneys, for reacher and stayer clusters respectively (see also **Figure 7**).

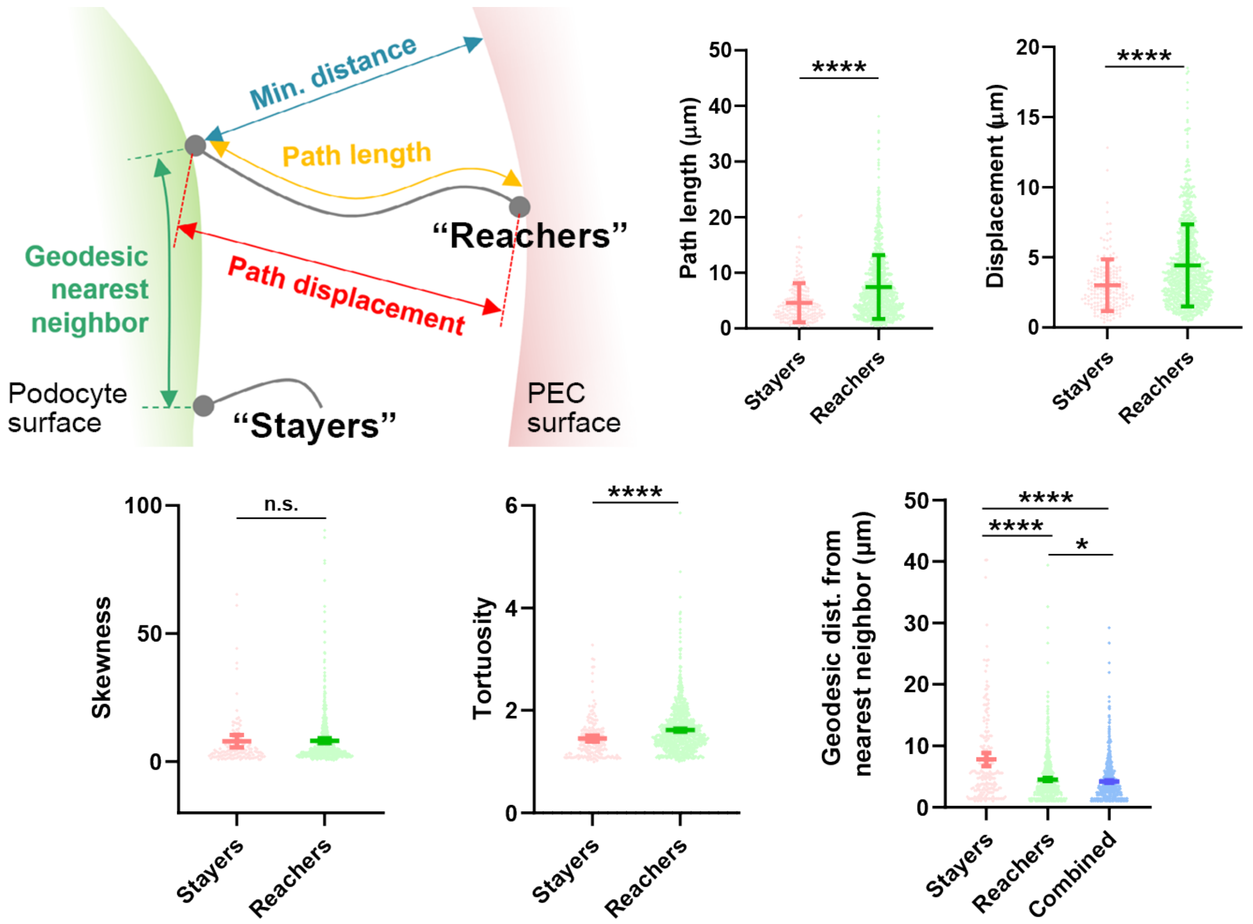

Supplementary Figure 28. Comparison of morphological and spatial parameters between stayers and reachers clusters obtained from 3D images of mouse kidneys (****: $p < 0.0001$).

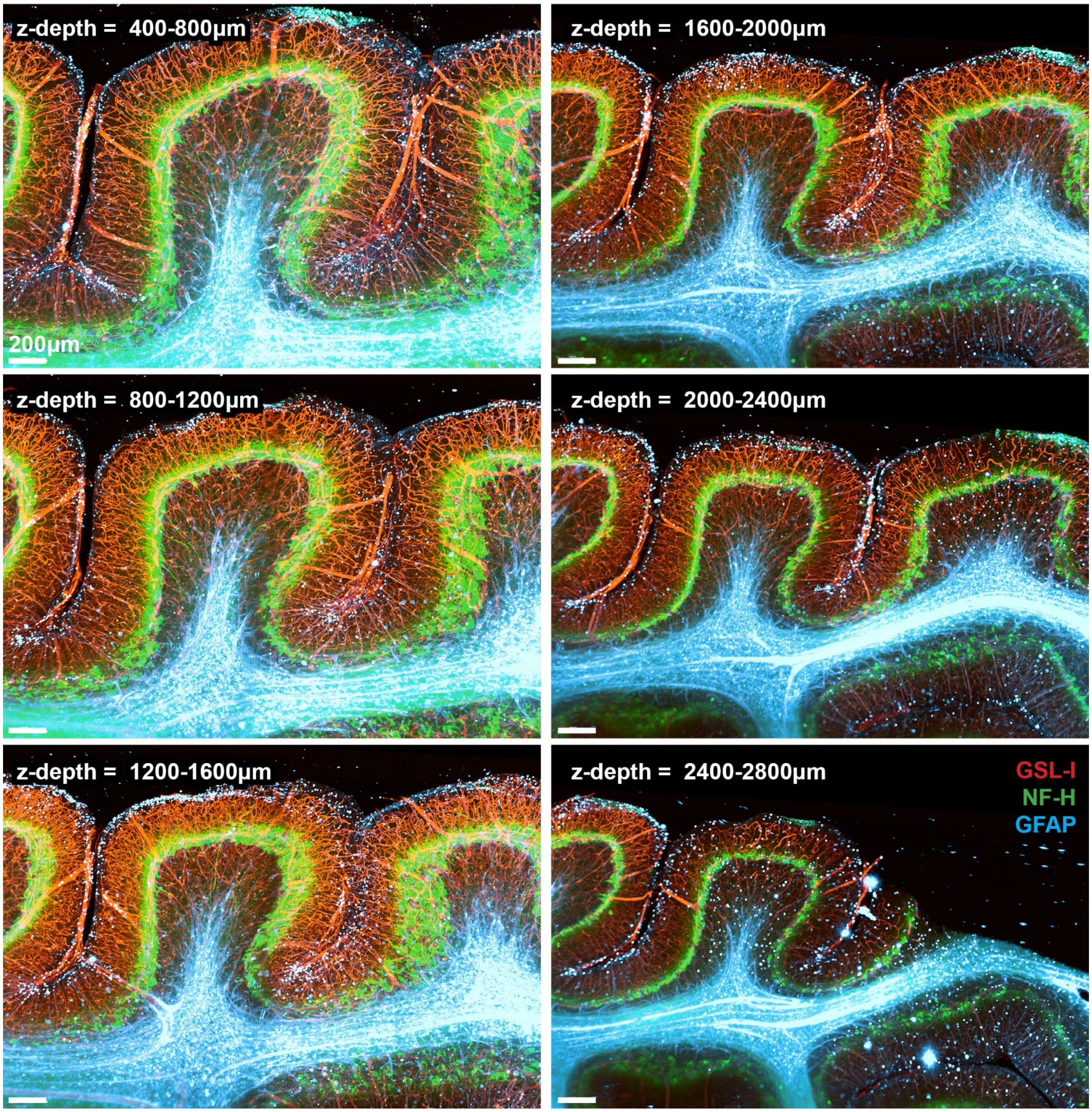

Supplementary Figure 29. Series of Z-projections of the 4mm × 8mm × 3mm human cerebellum triplex stained for vessels (with *Griffonia simplicifolia* lectin I, GSL-I, in red), neurofilaments (NF-H, in green), and astrocytes (GFAP, in blue).

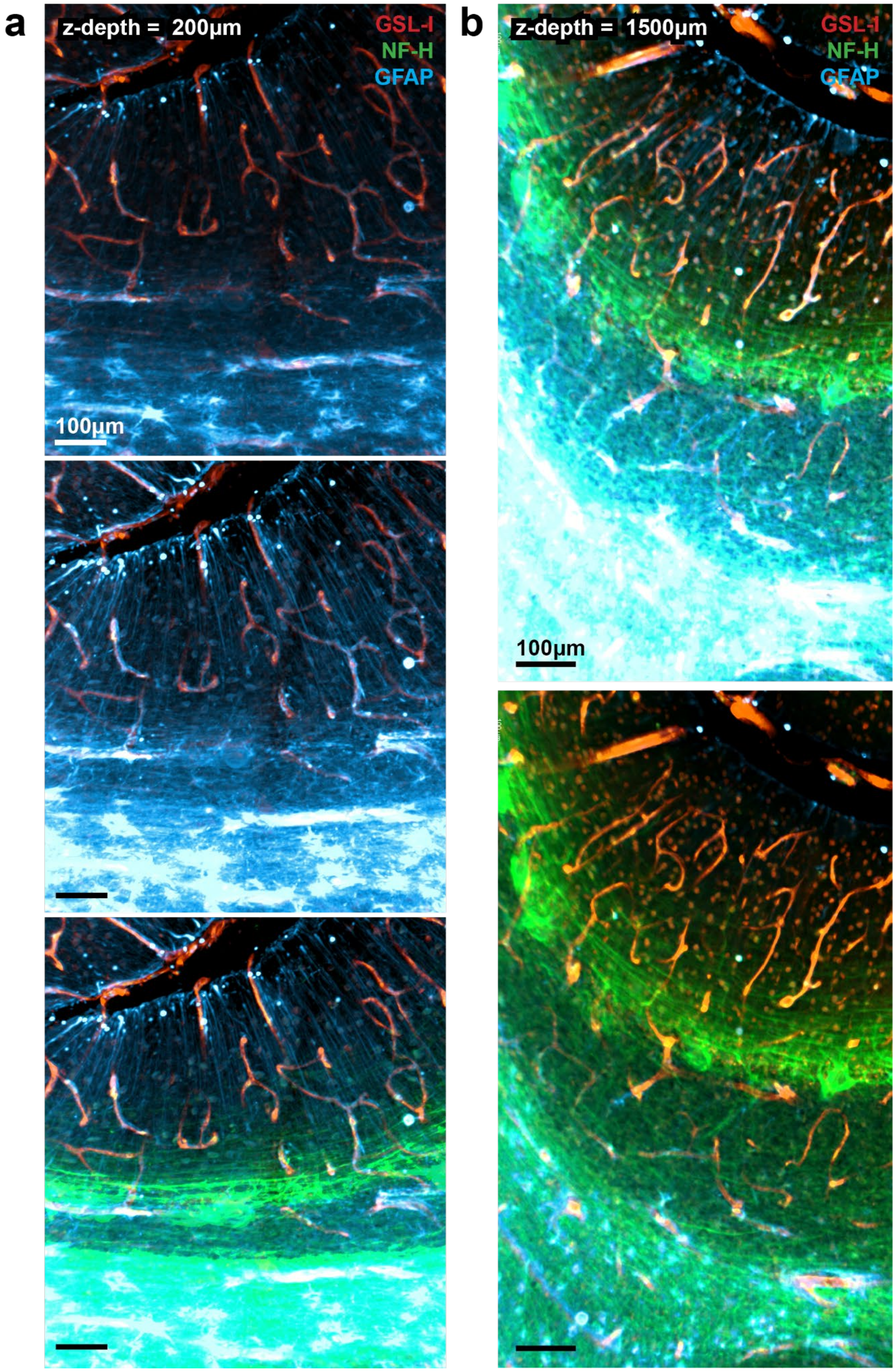

Supplementary Figure 30. High-resolution optical sections of the 3D cerebellum tissue volume. Zoomed-in view of the light-sheet microscopy image in **Figure 8**. Showing details such as astrocytic endfeets around blood vessels, Bergmann glia processes, and neurofilament fibres in all 3 layers at different *z*-depths. The images were rendered in different dynamic ranges to facilitate visualization of one layer over the other.

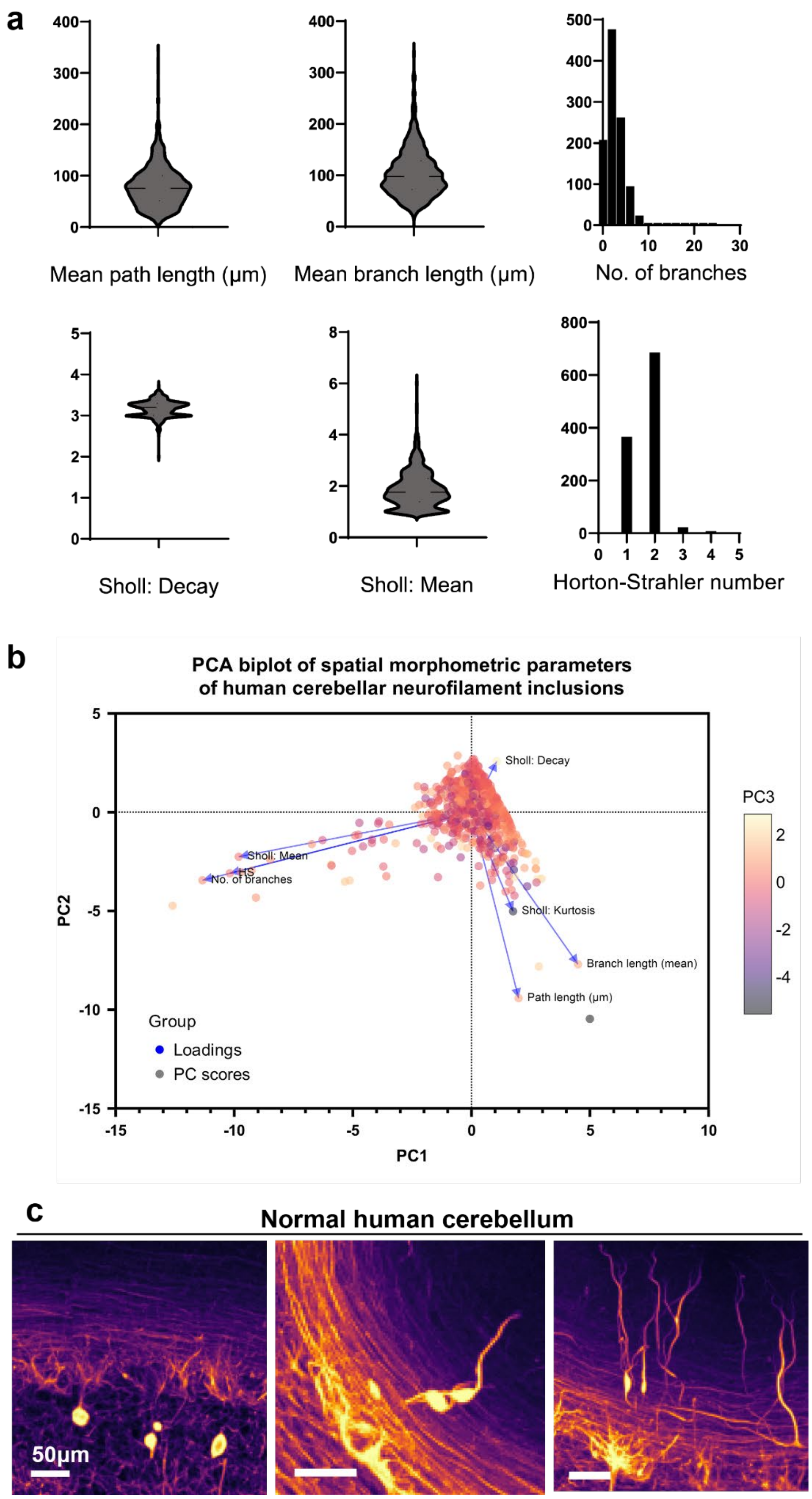

Supplementary Figure 31. Morphometric analysis and preliminary characterisation of human cerebellar NF-H-intense inclusions. a Descriptive statistics of all filaments in the 1 sample from **Fig. 8**. **b** principle component analysis with selected parameters (see also **Figure 8**). **c** Examples of similar inclusions are found in normal human cerebellar tissues.

Supplementary Tables

Supplementary Table 1. Antibody penetration exponential decay constant of benchmarking experiment using mouse hemi-brains as an estimate of signal inhomogeneity.

	Inhomogeneity, $\tau \times 10^3$ (confidence interval)
**INSIHGT**	0.301 (0.27, 0.33)
**CUBIC-HV**	1.11 (1.01, 1.22)
**eFLASH**	17.4 (18.6, 16.3)
**iDISCO**	6.83 (8.59, 5.07)
**SHANEL**	2.20 (2.29, 2.10)
**SWITCH-mELAST**	3.65 (2.91, 4.39)

Supplementary Table 2. List of antibodies tested with INSIHGT. Abbreviations for species: Ap: alpaca, Ck: chicken, Gp: guinea pig, Gt: goat, Ms: mouse, Hu: human, Rb: rabbit, Rt: rat, Sh: sheep.

Supplier	Cat. no.	Antigen	Host	Tested tissue(s)	Compatible
Santa cruz	sc-365529	11bHSD2	Ms	Ms brain	No
Abclonal	A12391	AIF1/IBA1	Rb	Ms brain	No
DSHB	N106/65	ANKG	Ms	Ms brain	No
Abclonal	A2887	AQP4	Rb	Ms brain	No
Abclonal	A12289	beta-tubulin	Rb	Ms brain	No
Abclonal	A18275	CARTPT	Rb	Ms brain	No
SantaCruz	sc-166940	c-Fos	Ms	Ms brain	No
DSHB	K55/7	ChIP1	Ms	Ms brain	No
Aves	CNP	CNP1	Ck	Ms brain	No
Abclonal	A18028	Cry1	Rb	Ms brain	No
Abclonal	A3019	CSF1R	Rb	Ms brain	No
Abcam	ab216588	Cux2	Rb	Ms brain	No
Sigma	HPA002130	DBH	Rb	Ms brain	No
Sigma	HPA001733	DLG3	Rb	Ms brain	No
Abclonal	A12930	DRD2	Rb	Ms brain	No
Abclonal	A1556	DRD3	Rb	Ms brain	No
DSHB	Mab3F52	GLP1R	Ms	Ms brain	No
DSHB	L21/32	GRIA2	Ms	Ms brain	No
Abclonal	A5465	HDC	Rb	Ms brain	No
Abcam	ab5076	Iba1	Gt	Ms brain	No
Abclonal	A0303	IGF1	Rb	Ms brain	No
Abcam	ab133474	LRRK2	Rb	Ms kidney	No
Abclonal	A1664	MBP	Rb	Ms brain	No
SYSY	260 308	NET/SLC6A2	Gp	Ms brain	No
AbClonal	A3048	NPHS1	Rb	Ms kidney	No
Abcam	ab50339	NPHS2	Rb	Ms kidney	No
Abclonal	A12814	Olig2	Rb	Ms brain	No
Abclonal	A20009	PLP1	Rb	Ms brain	No
Sigma	P0372	Podocin	Rb	Ms kidney	No
Abclonal	A14171	SERT/SLC6A4	Rb	Hu brain	No
Abclonal	A2799	SLC18A2/VMAT2	Rb	Ms brain	No
DSHB	PCRP-SOX2-1B3	SOX2	Ms	Ms brain	No
Sigma	AMab91108	TPH2	Ms	Hu brain	No
Abclonal	A14472	TRH	Rb	Ms brain	No
Sigma	AMAB91081	VGLUT2	Ms	Hu brain	No
Sigma	S5545	5-HT	Rb	Ms brain	Yes
Immusmol	IS040	5-HT	Ms	Ms brain	Yes
Abclonal	A7255	Acetyl-Histone-H3-K9	Rb	Ms testis	Yes

Abclonal	A2525	ADAMTS4	Rb	Ms brain	Yes
Abclonal	A1471	ADRA1A	Rb	Ms brain	Yes
Abclonal	A2809	ADRA2A	Rb	Ms brain	Yes
Abclonal	A16334	ADRB1	Rb	Ms brain	Yes
Abclonal	A2048	ADRB2	Rb	Ms brain	Yes
Abclonal	A12192	AGMAT	Rb	Ms brain	Yes
Abclonal	A8044	AGRP	Rb	Ms brain	Yes
Abclonal	A15637	Agt	Rb	Ms brain	Yes
Abclonal	A12326	Agt	Rb	Ms brain	Yes
Rockland	600-101-HB65	ALDH1L1	Gt	Ms brain	Yes
Abcam	ab56777	ALDH1L1	Ms	Ms brain	Yes
Proteintech	17390-1-AP	ALDH1L1	Rb	Ms brain	Yes
SYSY	278 102	ALDH1L1	Rb	Ms brain	Yes
Abclonal	A11618	ALDOC	Rb	Ms brain	Yes
SYSY	464 004	Aldolase C	Gp	Ms brain	Yes
Abclonal	A13354	Alpha synuclein	Rb	Hu brain	Yes
Abclonal	A20407	Alpha synuclein	Rb	Hu brain	Yes
BD	610787	Alpha synuclein	Ms	Hu brain	Yes
Abcam	ab184674	Alpha synuclein pS129	Ms	Hu brain	Yes
SYSY	386 006	Ankyrin G	Ck	Ms brain	Yes
Abclonal	A4195	AQP1	Rb	Ms kidney	Yes
Abclonal	A16209	AQP2	Rb	Ms kidney	Yes
SYSY	429 006	AQP4	Ck	Ms brain	Yes
SYSY	429 004	AQP4	Gp	Hu brain	Yes
Alomone Labs	AQP-005	AQP5	Rb	Ms lacrimal gland	Yes
Santa cruz	sc-17839	Arc	Ms	Ms brain	Yes
Synaptic systems	156 003	Arc	Rb	Ms brain	Yes
Abclonal	A1223	ARHGAP25	Rb	Ms brain	Yes
Invitrogen	MA1-06110	aSMA	Ms	Ms & Hu kidney	Yes
Abclonal	A17910	aSMA	Rb	Ms kidney	Yes
Progen	690001	aSMA	Rb	Ms & Hu kidney	Yes
Abclonal	A11217	ATP5A1	Rb	Ms kidney	Yes
Abclonal	A4873	BDNF	Rb	Ms brain	Yes
Abcam	ab78078	bIIITub	Ms	Hu brain	Yes
Abcam	ab11862	C3	Rt	Ms brain	Yes
Abclonal	A1212	CA3	Rb	Ms brain	Yes
SYSY	214 011	Calbindin	Ms	Ms brain	Yes
AbClonal	A4284	Calbindin	Rb	Ms brain	Yes
CST	13176S	Calbindin	Rb	Ms brain	Yes
Swant	CB300	Calbindin D28K	Ms	Ms brain	Yes
Abclonal	A11979	CALCRL	Rb	Ms brain	Yes

R&D	AF5065	Calretinin	Gt	Ms brain	Yes
Abclonal	A0198	CaMKII	Rb	Ms brain	Yes
Origene	TA808345	CaMKIIb	Ms	Ms brain	Yes
DSHB	PCRP-CAMKK2-1H7	CAMKK2b	Ms	Ms brain	Yes
DSHB	K65/35-s	CASPR/Neurexin IV	Ms	Ms brain	Yes
SYSY	258 106	CB1R	Ck	Ms brain	Yes
SYSY	438 004	CCK	Gp	Ms brain	Yes
Abclonal	A1759	CCK	Rb	Ms brain	Yes
SYSY	HS-360 117	CD4	Rt	Ms spleen	Yes
SYSY	HS-384 117	CD11b/ITGAM	Rt	Ms colon	Yes
Abclonal	A1508	CD11c/ITGAX	Rb	Ms colon	Yes
Abclonal	A5662	CD13	Rb	Ms brain	Yes
Abclonal	A11669	CD13	Rb	Ms brain	Yes
Jotbody	AP01388a	CD19	Rb	Ms spleen	Yes
Abclonal	A12711	CD133	Rb	Ms kidney	Yes
Abclonal	A4900	CD31-PECAM1	Rb	Ms kidney, brain	Yes
Abclonal	A0761	CD34	Rb	Ms kidney	Yes
LSBio	LS-C359273	CD44	Ms	Ms kidney	Yes
Abclonal	A15995	CENP-A	Rb	Ms testis	Yes
Abclonal	A16641	c-Fos	Rb	Ms brain	Yes
Abclonal	A0236	c-Fos	Rb	Ms brain	Yes
CST	2250S	c-Fos	Rb	Ms brain	Yes
SYSY	226 017	c-Fos	Rt	Ms brain	Yes
Abclonal	A5542	CGRP	Rb	Ms brain	Yes
Millipore	AB144	ChAT	Gt	Ms brain	Yes
Invitrogen	MA3-31382	ChAT	Ms	Ms brain	Yes
Abclonal	A13244	ChAT	Rb	Ms brain	Yes
SYSY	297 013	ChAT	Rb	Ms brain	Yes
DSHB	K60/73	ChIP2	Ms	Ms brain	Yes
DSHB	K66/38	ChIP3	Ms	Ms brain	Yes
SYSY	223 017	CHRM2	Rt	Ms brain	Yes
Abclonal	A1602	CHRM3	Rb	Ms brain	Yes
Abclonal	A3792	CLCNKA	Rb	Ms kidney	Yes
SYSY	355 006	CNP1	Ck	Ms brain	Yes
SYSY	355 004	CNP1	Gp	Ms brain	Yes
Abclonal	A20483	CTIP/BCL11B	Rb	Ms brain	Yes
Abclonal	A2160	CYP2E1	Rb	Ms liver	Yes
Abclonal	A2544	CYP3A4	Rb	Ms liver	Yes
SYSY	382 003	DARPP32	Rb	Ms brain	Yes
Millipore	MAB369	DAT	Rb	Ms brain	Yes
SYSY	284 006	DAT	Ck	Ms brain	Yes
SYSY	284 003	DAT/SLC6A3	Rb	Ms brain	Yes
Abcam	ab189991	DBH	Gt	Ms brain	Yes
Abclonal	A13971	DBH	Rb	Ms brain	Yes

Invitrogen	PA3-925	DBH	Sh	Ms brain	Yes
Abclonal	A3828	DDC	Rb	Ms brain	Yes
Sigma	HPA017742	DDC	Rb	Ms brain	Yes
Abcam	ab167612	DeltaNp63	Ms	Hu colon cancer	Yes
Immusmol	IS1005	Dopamine	Rb	Ms brain	Yes
Abclonal	A2893	DRD1	Rb	Ms brain	Yes
SYSY	376 205	DRD2	Gp	Ms brain	Yes
Millipore	205604	E-Cadherin	Rt	Ms lacrimal gland	Yes
Abcam	ab41621	EAAT2	Rb	Ms brain	Yes
Abclonal	A2722	EGR1	Rb	Ms brain	Yes
Abcam	ab204991	ELAVL2	Rb	Ms brain	Yes
Invitrogen	MA5-26826	FEZF2	Ms	Ms brain	Yes
Abcam	ab205606	Flag	Rb	Ms brain	Yes
DSHB	PCRP-FOXA2-3A2	FOXA2	Ms	Ms brain	Yes
DSHB	PCRP-FOXP4-1G7	FOXP4	Ms	Ms brain	Yes
Immusmol	IS1036	GABA	Ck	Ms brain	Yes
Sigma	A2052	GABA	Rb	Ms brain	Yes
Sigma	HPA055746	GABARA1	Rb	Ms brain	Yes
SYSY	322 102	GABBR1	Rb	Ms brain	Yes
SYSY	322 203	GABBR2	Rb	Ms brain	Yes
SYSY	224 205	GABRA1	Gp	Ms brain	Yes
SYSY	224 103	GABRA2	Rb	Ms brain	Yes
SYSY	224 303	GABRA3	Rb	Ms brain	Yes
DSHB	GABRA4-R24	GABRA4	Rb	Ms brain	Yes
SYSY	224 503	GABRA5	Rb	Ms brain	Yes
SYSY	224 603	GABRA6	Rb	Ms brain	Yes
SYSY	224 703	GABRB1	Rb	Ms brain	Yes
SYSY	224 803	GABRB2	Rb	Ms brain	Yes
SYSY	224 403	GABRB3	Rb	Ms brain	Yes
SYSY	224 003	GABRG2	Rb	Ms brain	Yes
DSHB	GABRG3-R21	GABRG3	Rb	Ms brain	Yes
Abcam	ab111070	GAD1/2	Rb	Ms brain	Yes
Abclonal	A0971	GAD2	Rb	Ms brain	Yes
Abclonal	A14609	GCG	Rb	Ms brain, pancreas	Yes
SantaCruz	sc-25311	Gephyrin	Ms	Ms & Hu brain	Yes
Aves	GFAP	GFAP	Ck	Ms brain	Yes
Dako	M0761	GFAP	Ms	Hu brain	Yes
Invitrogen	13-0300	GFAP	Rt	Ms & Hu brain	Yes
Abcam	ab252881	GFP	Gt	Ms brain	Yes
Abcam	ab6673	GFP	Rt	Ms brain	Yes
SYSY	N0304	GFP	Ap	Ms brain	Yes

Invitrogen	PA5-47769	GFRAL	Sh	Ms brain	Yes
Abclonal	A4981	GLP1	Rb	Ms brain, pancreas	Yes
DSHB	Mab7F38	GLP1R	Ms	Ms brain	Yes
Abclonal	A8547	GLP1R	Rb	Ms brain	Yes
SYSY	367 005	GLUL	Gp	Ms liver	Yes
SYSY	367 011	GLUL	Ms	Ms liver	Yes
Immusmol	IS1001	L-Glutamate	Rb	Ms brain	Yes
SYSY	456 003	Glutaminase 1	Rb	Ms brain	Yes
Immusmol	IS1034	Glycine	Rb	Ms brain	Yes
Abclonal	A11408	GM130	Rb	Ms kidney	Yes
Abclonal	A5625	GNRH1	Rb	Ms brain	Yes
SYSY	182 011	GRIA1	Ms	Ms brain	Yes
AbClonal	A1826	GRIA1	Rb	Ms brain	Yes
SYSY	182 111	GRIA2	Ms	Ms brain	Yes
SYSY	182 203	GRIA3	Rb	Ms brain	Yes
SYSY	182 303	GRIA4	Rb	Ms brain	Yes
SYSY	180 313	GRIK1	Rb	Ms brain	Yes
AbClonal	A9722	GRIK2	Rb	Ms brain	Yes
SYSY	180 003	GRIK2	Rb	Ms brain	Yes
SYSY	180 203	GRIK3	Rb	Ms brain	Yes
SYSY	180 103	GRIK5	Rb	Ms brain	Yes
SYSY	114 011	GRIN1	Ms	Ms brain	Yes
DSHB	327/95	GRIN2A	Ms	Ms brain	Yes
SYSY	244 003	GRIN2A/B	Rb	Ms brain	Yes
SYSY	244 115	GRIN2B	Gp	Ms brain	Yes
DSHB	N59/36	GRIN2B	Ms	Ms brain	Yes
AbClonal	A3056	GRIN2B	Rb	Ms brain	Yes
SYSY	191 103	GRM2	Rb	Ms brain	Yes
SYSY	191 203	GRM7	Rb	Ms brain	Yes
AbClonal	A2964	GRM8	Rb	Ms brain	Yes
Abclonal	A7253	H3K27ac	Rb	Ms testis	Yes
Abclonal	A2361	H3K27me	Rb	Ms testis	Yes
Abclonal	A2362	H3K27me2	Rb	Ms testis	Yes
Abclonal	A2363	H3K27me3	Rb	Ms testis	Yes
Abclonal	A7255	H3K9Ac	Rb	Ms testis	Yes
Abclonal	A3156	H3R8me	Rb	Ms testis	Yes
Abclonal	A3157	H3R8me2-asymmetric	Rb	Ms testis	Yes
Abclonal	A21207	H3R8me2-symmetric	Rb	Ms testis	Yes
DSHB	N70/28	HCN1	Ms	Ms brain	Yes
DSHB	N114/10	HCN4	Ms	Ms brain	Yes
Immunsmol	IS1039	Histamine	Rb	Ms brain	Yes
Abclonal	A3692	Histone H2A	Rb	Ms brain, testis	Yes
Abclonal	A17813	Histone H2A.V	Rb	Ms testis	Yes
Abclonal	A12442	Histone H2A.Z	Rb	Ms testis	Yes

Abclonal	A11540	Histone H2AX	Rb	Ms testis	Yes
Abclonal	A4835	Histone H3.3	Rb	Ms testis	Yes
Abclonal	A11252	HLA-DQA1	Rb	Ms spleen, colon	Yes
Abclonal	A8077	HSD11B2	Rb	Ms brain	Yes
Abclonal	A2801	HTR1A	Rb	Ms brain	Yes
Abclonal	A18285	HTR1B	Rb	Ms brain	Yes
Abclonal	A14743	HTR1F	Rb	Ms brain	Yes
Abclonal	A20538	HTR2A	Rb	Ms brain	Yes
Immunostar	24288	HTR2A	Rb	Ms brain	Yes
AbClonal	A5647	HTR3A	Rb	Ms brain	Yes
Abclonal	A2802	HTR4	Rb	Ms brain	Yes
Abclonal	A19706	HTR7	Rb	Ms brain	Yes
SYSY	234 009	Iba1	Ck	Ms brain	Yes
Wako	019-19741	Iba1	Rb	Ms brain	Yes
SYSY	117 002	IP3R type 1	Rb	Ms brain	Yes
R&D	AF599	Jagged1	Gt	Ms & Hu brain	Yes
DSHB	L18A/3	KCNMB4	Ms	Ms brain	Yes
Abclonal	A17061	KEAP1	Rb	Ms brain	Yes
DSHB	N112B/14	Kir2.1	Ms	Ms brain	Yes
Abclonal	A1024	KRT8/TROMA-1	Rb	Ms skin	Yes
DSHB	SP2/0	KRT8/TROMA-1	Rt	Ms skin	Yes
Abclonal	A0247	KRT19	Rb	Ms liver	Yes
DSHB	K36/15	Kv1.1	Ms	Ms brain	Yes
DSHB	K14/16	Kv1.2	Ms	Ms brain	Yes
DSHB	K89/34	Kv2.1	Ms	Ms brain	Yes
DSHB	K37/89	Kv2.2	Ms	Ms brain	Yes
DSHB	K57/1	Kv4.2	Ms	Ms brain	Yes
DSHB	K9/40	Kvb1.1	Ms	Ms brain	Yes
DSHB	K17/70	Kvb2	Ms	Ms brain	Yes
AbClonal	A19970	Laminin b1	Rb	Ms kidney	Yes
Abclonal	A2999	LEPR	Rb	Ms brain	Yes
Abclonal	A7045	macroH2A.1	Rb	Ms testis	Yes
Abclonal	A2572	MAP2	Rb	Ms brain	Yes
SYSY	188 011BT	MAP2	Ms	Ms brain	Yes
Abcam	ab24233	MC4R	Rb	Ms brain	Yes
Abclonal	A10228	MC4R	Rb	Ms brain	Yes
Abclonal	A3013	MCT1/SLC16A1	Rb	Ms brain & colon	Yes
AbClonal	A3673	mGluR2	Rb	Ms brain	Yes
Abcam	ab206486	Myc	Rt	Ms brain	Yes
DSHB	PCRP-MYCN-1A9	MYCN	Ms	Ms brain	Yes
AbClonal	A11683	NaK-ATPase	Rb	Ms kidney	Yes
DSHB	K69/3	Nav1.2	Ms	Ms brain	Yes
DSHB	K87A/10	Nav1.6	Ms	Ms brain	Yes

Abclonal	A1421	NET/SLC6A2	Rb	Ms brain	Yes
SYSY	260 008	NET/SLC6A2	Rb	Ms brain	Yes
Abcam	ab104224	NeuN	Ms	Ms & Hu brain	Yes
Abclonal	A19086	NeuN	Rb	Ms brain	Yes
AbClonal	A19084	Neurofilament-H	Rb	Ms & Hu brain	Yes
Sigma	N4142	Neurofilament-H	Rb	Ms & Hu brain	Yes
AbClonal	A0257	Neurofilament-L	Rb	Ms brain	Yes
AbClonal	A19085	Neurofilament-M	Rb	Ms brain	Yes
Abclonal	A12326	Neurotensin	Rb	Ms brain	Yes
AbClonal	A11699	NMDAR1	Rb	Ms brain	Yes
AbClonal	A0924	NMDAR2A	Rb	Ms brain	Yes
SYSY	432 006	nNOS	Ck	Ms brain	Yes
SYSY	432 005	nNOS	Gp	Ms brain	Yes
Abclonal	A8319	NOL3/Arc	Rb	Ms brain	Yes
Immusmol	IS1042	L-Noradrenaline	Rb	Ms brain	Yes
Invitrogen	PA5-39300	NPAS4	Rb	Ms brain	Yes
SYSY	394 006	NPY	Ck	Ms brain	Yes
Abclonal	A3178	NPY	Rb	Ms brain	Yes
DSHB	PCRP-NRF1-3D4	NRF1	Ms	Ms brain	Yes
Abclonal	AP1133	NRF2-pS40	Rb	Ms brain	Yes
AbClonal	A2029	NT5E	Rb	Ms liver	Yes
Abclonal	A12326	NTS	Rb	Ms brain	Yes
Millipore	AB3704	Orexin A	Rb	Ms brain	Yes
Jotbody	0112	p53	Ap	Hu colon	Yes
DSHB	N419/40	Pan-Na.v1	Ms	Ms brain	Yes
Abcam	ab18257	PARK7	Rb	Ms brain	Yes
Abcam	ab179812	Parkin	Rb	Ms kidney	Yes
DSHB	PCRP-PAX2-1A7	Pax2	Ms	Ms brain	Yes
SYSY	153 011	Pax6	Ms	Ms brain	Yes
SYSY	480 004	PCP4	Gp	Ms brain	Yes
AbClonal	A2103	PDGFRA	Rb	Ms kidney	Yes
AbClonal	A19531	PDGFRB	Rb	Ms kidney	Yes
Abclonal	AP1022	PDH-pS293	Rb	Ms brain	Yes
CST	37115	PDH-pS293	Rb	Ms brain	Yes
Jotbody	0002	PD-L1	Ap	Hu colon cancer	Yes
Abclonal	A5830	PDYN	Rb	Ms brain	Yes
Abclonal	AP0687	Phospho-Histone-H2AX-S139	Rb	Ms testis	Yes
Abclonal	AP0093	Phospho-Histone-H3-T11	Rb	Ms testis	Yes
CST	4370S	Phospho-p44/42 MAPK	Rb	Ms brain	Yes
Invitrogen	44-923G	Phospho-	Rb	Ms brain	Yes

		S6(S244,S247)			
Abclonal	A9655	Phox2b	Rb	Ms brain	Yes
Abcam	ab186303	PINK1	Ms	Ms kidney	Yes
Abclonal	A10200	PODXL	Rb	Ms kidney	Yes
NeuroMab	75-028	PSD95	Ms	Ms brain	Yes
SYSY	195 004	PVALB	Gp	Ms brain	Yes
Abcam	ab32895	PVALB	Gt	Ms brain	Yes
Abclonal	A19098	PVALB	Rb	Ms brain	Yes
Invitrogen	PA1-933	PVALB	Rb	Ms & Hu brain	Yes
Abclonal	A19710	SOX9	Rb	Ms testis	Yes
Abclonal	A10029	RBP1	Rb	Ms liver	Yes
Abclonal	A17936	RELN	Rb	Ms brain	Yes
Abclonal	A1585	Renin	Rb	Ms kidney	Yes
Abcam	ab125244	RFP	Ms	Ms brain & kidney	Yes
Abclonal	A19753	RORbeta	Rb	Ms brain	Yes
Enzo	ENZ-ABS307-0100	S100b	Rb	Ms brain	Yes
DSHB	N19/2	SAP102	Ms	Ms brain	Yes
SYSY	327 003	SATB2	Rb	Ms brain	Yes
Abclonal	A15097	scnn1g	Rb	Ms kidney	Yes
SYSY	340 004	SERT	Gp	Ms brain	Yes
SYSY	340 004	SERT/SLC6A4	Gp	Ms brain	Yes
Abclonal	A12999	slc12a1	Rb	Ms kidney	Yes
Abclonal	A18382	slc12a3	Rb	Ms kidney	Yes
Abclonal	A15991	slc14a1	Rb	Ms kidney	Yes
Abclonal	A13208	slc14a2	Rb	Ms kidney	Yes
Abclonal	A15177	SLC17A6 (VGLUT2)	Rb	Ms brain	Yes
Abclonal	A12879	SLC17A7	Rb	Ms brain	Yes
Abclonal	A2799	SLC18A2	Rb	Ms brain	Yes
LSBio	LS-B6724	SLC1A2	Ms	Ms brain	Yes
Abclonal	A1814	SLC22A6	Rb	Ms kidney	Yes
Abclonal	A20271	SLC5A2	Rb	Ms kidney	Yes
Invitrogen	PA5-101896	SLC5A3	Rb	Ms kidney	Yes
Abclonal	A17532	SLC9A3	Rb	Ms kidney	Yes
AbClonal	A19576	SOD2	Rb	Ms kidney	Yes
Abcam	ab97959	SOX2	Rb	Ms brain	Yes
SYSY	347 003	SOX2	Rb	Ms brain	Yes
SYSY	366 006	SST	Ck	Ms brain	Yes
AbClonal	A9274	SST	Rb	Ms brain	Yes
Millipore	MAB354	SST	Rt	Ms brain	Yes
R&D	MAB2358	SST	Gt	Ms brain	Yes
Novus	NB300-104	Synapsin I	Rb	Ms brain	Yes
Abclonal	A13550	Tac1	Rb	Ms brain	Yes
Abclonal	A6312	TAC3	Rb	Ms brain	Yes
Abcam	A121690	tdTomato	Gt	Ms brain	Yes

Abclonal	A5865	TFRC	Rb	Ms kidney	Yes
SYSY	213 104	TH	Gp	Ms brain	Yes
Abclonal	A0028	TH	Rb	Ms brain	Yes
Millipore	AB152	TH	Rb	Ms brain	Yes
SYSY	400 006	TMEM119	Gp	Ms brain	Yes
Abcam	ab209064	TMEM119	Rb	Ms brain	Yes
Abclonal	A7147	TPH2	Rb	Ms brain	Yes
Abclonal	A10482	TREM2	Rb	Ms brain	Yes
DSHB	N67/15	TRPC5	Ms	Ms brain	Yes
Novus Biologicals	NBP1-80651	UBAP1	Rb	Ms brain	Yes
Abclonal	A1920	UMOD	Rb	Ms kidney	Yes
Abclonal	A19707	VDAC1	Rb	Ms kidney	Yes
Abcam	ab33168	VE-Cadherin	Rb	Ms kidney	Yes
Abclonal	A19607	Vimentin	Rb	Ms kidney	Yes
SYSY	443 005	VIP	Gp	Ms brain	Yes
AbClonal	A12531	VIP	Rb	Ms brain	Yes
Bioss	bs-0077R	VIP	Rb	Ms brain	Yes
AbClonal	A1335	VWF	Rb	Ms kidney	Yes
SYSY	362 003	ZBTB20	Rb	Ms brain	Yes

Supplementary Table 3. All tested protein-conjugating fluorophores and their compatibility with INSIHGT. For the excitation peaks, absorbance peaks were provided if the excitation peaks were unknown or not provided in the literature.

Fluorophore name	Excitation/Emission peak (nm)	Compatibility with INSIHGT
Alexa Fluor 405	401 / 421	Yes
Alexa Fluor 488	499 / 520	Yes
Atto 430LS	435 / 545	Yes
Fluorescein	498 / 516	Yes
Atto 488	499 / 520	Yes
Dylight 488	493 / 520	Yes
Atto 490LS	495 / 658	Yes
iFluor 488	490 / 520	Yes
Alexa Fluor 514	518 / 542	Yes
Alexa Fluor 532	534 / 553	Yes
Alexa Fluor 555	553 / 568	Yes
Atto 550	554 / 576	Yes
iFluor 546	541 / 557	Yes
Alexa Fluor 546	561 / 573	Yes
Alexa Fluor 568	578 / 603	Yes
Tetramethylrhodamine	552 / 577	Yes
Alexa Fluor 594	590 / 618	Yes
Dylight 594	593 / 618	Yes
Alexa Fluor 647	650 / 671	Yes
Atto 647N	645 / 664	Yes
Cy5	646 / 667	Yes
Cy5.5	674 / 692	Yes
SeTau-647	648 / 694	Yes
Dylight 649	651 / 672	Yes
iFluor 647	655 / 671	Yes
Alexa Fluor 680	681 / 704	Yes
Atto 700	700 / 716	Yes
Alexa Fluor 700	696 / 720	Yes
iFluor 700	690 / 710	Yes
Dylight 700	707 / 728	Yes

Supplementary Table 4. Basic image acquisition parameters for all microscopy images in this study. For confocal microscopy, the objectives used are Leica HC PL APO ×10/0.40 CS2 or Leica HC PL APO 40X/1.30 Oil CS2. For MesoSPIM light-sheet microscopy, the detection was done using an Olympus MVX-ZB10 Zoombody and only the magnification during imaging was provided. For two-photon tomography, a Nikon CFI75 LWD 16X W (NA 0.8) objective was used. The "S" prefix in the Figure refers to supplementary figures.

Figure	Microscopy	Excitation wavelength(s) (nm)	Objective Magnification/ Numerical aperture
2B	Leica SP8 confocal	488, 649	10x/ 0.40
3B	MesoSPIM v5.1	633	1.6x
3C-D	MesoSPIM v5.1	488, 561, 633	1.6x
3E-I	MesoSPIM v5.1	488, 561, 633	1.6x
3J-L	Two-photon tomography	780	16x/ 0.8
4B	Leica SP8 confocal	405, 649	40x/ 1.30
4C	Leica SP8 confocal	649	40x/ 1.30
4D	Leica SP8 confocal	405, 488, 561, 649	40x/ 1.30
4H	Leica SP8 confocal	405, 488, 561, 649	40x/ 1.30
5B-5F	Leica SP8 confocal	405, 488, 649	10x/ 0.40
5H-I	Two-photon tomography	780	16x/ 0.8
6B-C, G, I	Leica SP8 confocal	405, 488, 561, 649	10x/ 0.40
7A-D	Leica SP8 confocal	405, 488, 561, 649	40x/ 1.30
8A-C, 8F	MesoSPIM v5.1	488, 561, 633	1.6x
8D, E	Leica SP8 confocal	488, 561, 649	40x/ 1.30
S1A-B	Leica SP8 confocal	405, 649	40x/ 1.30
S2	MesoSPIM v5.1	488, 561	1.6x
S3B	Leica SP8 confocal	405, 649	40x/ 1.30

S4B	Leica SP8 confocal	488, 649	10x/ 0.40
S6A	Leica SP8 confocal	488, 649	10x/ 0.40
S7A	Leica SP8 confocal	488	40x/ 1.30
S8	MesoSPIM v5.1	488	1.6x
S9A-C	Two-photon tomography	780	16x/ 0.8
S10-S12	Leica SP8 confocal	405, 488, 561, 649	40x/ 1.30
S13	Leica SP8 confocal	488, 649	40x/ 1.30
S15	Two-photon tomography	780	16x/ 0.8
S16	Leica SP8 confocal	405, 488, 561, 649	40x/ 1.30
S17A	Leica SP8 confocal	488, 649	10x/ 0.40
S17B	Leica SP8 confocal	488, 649	40x/ 1.30
S26	MesoSPIM v5.1	488, 561, 633	3.2x
S27	MesoSPIM v5.1	488, 561, 633	3.2x

Supplementary Table 5. Basic physical chemistry characteristics of $[B_{12}H_{12}]^{2-}$ [9,14]

Ion radius	400 pm
$T\Delta S_{struct,water}$	14.8 kcal mol^{-1}
ΔHB	-2.31
ΔG_{hyd}	-140 kcal mol^{-1}
ΔH_{hyd}	-145
$T\Delta S_{hyd}$	-5 kcal mol^{-1}
Polarizability	25.7 Å^3 (calculated)
Hydration Δr	9.6 pm (calculated)
Average number of H_2O molecules in the hydration shell	1.8 (calculated)
$\Delta_{hyd}S$	-17.9 cal K^{-1} mol^{-1}
K_a with αCD in water	100 L mol^{-1}
K_a with βCD in water	200 L mol^{-1}
K_a with γCD in water	2,000 L mol^{-1}

Supplementary References

1. Jacso, T. *et al.* The Mechanism of Denaturation and the Unfolded State of the α-Helical Membrane-Associated Protein Mistic. (2013) doi:10.1021/ja408644f.
2. Ganguly, P., Boserman, P., van der Vegt, N. F. A. & Shea, J.-E. Trimethylamine N-oxide Counteracts Urea Denaturation by Inhibiting Protein–Urea Preferential Interaction. (2017) doi:10.1021/jacs.7b11695.
3. Riddlestone, I. M., Kraft, A., Schaefer, J. & Krossing, I. Taming the Cationic Beast: Novel Developments in the Synthesis and Application of Weakly Coordinating Anions. *Angew. Chem. Int. Ed.* **57**, 13982–14024 (2018).
4. Lipping, L. *et al.* Superacidity of closo-Dodecaborate-Based Brønsted Acids: a DFT Study. (2015) doi:10.1021/jp506485x.
5. Karki, K., Gabel, D. & Roccatano, D. Structure and dynamics of dodecaborate clusters in water. *Inorg. Chem.* **51**, 4894–4896 (2012).
6. Jiang, Y. *et al.* Highly Structured Water Networks in Microhydrated Dodecaborate Clusters. *J. Phys. Chem. Lett.* **13**, 11787–11794 (2022).
7. Jiang, Y. *et al.* Unraveling hydridic-to-protonic dihydrogen bond predominance in monohydrated dodecaborate clusters. *Chem. Sci.* **13**, 9855–9860 (2022).
8. Bukovsky, E. V. *et al.* Comparison of the Coordination of B12F122–, B12Cl122–, and B12H122– to Na+ in the Solid State: Crystal Structures and Thermal Behavior of Na2(B12F12), Na2(H2O)4(B12F12), Na2(B12Cl12), and Na2(H2O)6(B12Cl12). (2017) doi:10.1021/acs.inorgchem.6b02920.
9. Assaf, K. I. *et al.* Water Structure Recovery in Chaotropic Anion Recognition: High-Affinity Binding of Dodecaborate Clusters to γ-Cyclodextrin. *Angew. Chem. Int. Ed.* **54**, 6852–6856 (2015).
10. Goszczyński, T. M., Fink, K., Kowalski, K., Leśnikowski, Z. J. & Boratyński, J. Interactions of Boron Clusters and their Derivatives with Serum Albumin. *Sci. Rep.* **7**, 9800 (2017).

11. Murray, E. *et al.* Simple, Scalable Proteomic Imaging for High-Dimensional Profiling of Intact Systems. *Cell* **163**, 1500–1514 (2015).

12. Yau, C. N. *et al.* Principles of deep immunohistochemistry for 3D histology. *Cell Rep Methods* **3**, 100458 (2023).

13. Lai, H. M. *et al.* Antibody stabilization for thermally accelerated deep immunostaining. *Nat. Methods* **19**, 1137–1146 (2022).

14. Assaf, K. I. & Nau, W. M. The Chaotropic Effect as an Assembly Motif in Chemistry. *Angew. Chem. Int. Ed Engl.* **57**, 13968–13981 (2018)
