# Supplementary material for: INSIHGT: an accessible multi-scale, multi-modal 3D spatial biology platform
Source: Nat Commun. 2024 Dec 30;15:10888. doi: 10.1038/s41467-024-55248-0 (PMC11685604; doi:10.1038/s41467-024-55248-0)
Supplement: Supplementary file 2 — Description of Additional Supplementary Files [file 41467_2024_55248_MOESM2_ESM.pdf]

Supplementary Movies.

Supplementary Movie 1. 3D visualization of the whole mouse brain stained for calcium-binding proteins in **Fig. 3j-l.**

Supplementary Movie 2. Demonstration of a zoom-in from a centimeter to micrometer scale on a mesoscale-imaged human hemi-brainstem at micrometer resolution of immunostaining signals on phosphorylated alpha-synuclein at serine 129. This is the same specimen detailed in **Fig. 5g-i** and **Supplementary Fig. 19.**

Supplementary Movie 3. 3D visualization of the 3 mm-thick, 3-plex immunostained human cerebellum in **Fig. 8** and **Supplementary Fig. 29-31.**
