# Supplementary material for: INSIHGT: an accessible multi-scale, multi-modal 3D spatial biology platform
Source: Nat Commun. 2024 Dec 30;15:10888. doi: 10.1038/s41467-024-55248-0 (PMC11685604; doi:10.1038/s41467-024-55248-0)
Supplement: Supplementary file 6 — Reporting Summary [file 41467_2024_55248_MOESM6_ESM.pdf]

nature portfolio

Corresponding author(s): Hei Ming Lai

Last updated by author(s): Nov 2, 2024

Reporting Summary

Nature Portfolio wishes to improve the reproducibility of the work that we publish. This form provides structure for consistency and transparency in reporting. For further information on Nature Portfolio policies, see our Editorial Policies and the Editorial Policy Checklist.

Statistics

For all statistical analyses, confirm that the following items are present in the figure legend, table legend, main text, or Methods section.

n/a	Confirmed	
☐	☒	The exact sample size (*n*) for each experimental group/condition, given as a discrete number and unit of measurement
☐	☒	A statement on whether measurements were taken from distinct samples or whether the same sample was measured repeatedly
☐	☒	The statistical test(s) used AND whether they are one- or two-sided<br>*Only common tests should be described solely by name; describe more complex techniques in the Methods section.*
☒	☐	A description of all covariates tested
☐	☒	A description of any assumptions or corrections, such as tests of normality and adjustment for multiple comparisons
☐	☒	A full description of the statistical parameters including central tendency (e.g. means) or other basic estimates (e.g. regression coefficient) AND variation (e.g. standard deviation) or associated estimates of uncertainty (e.g. confidence intervals)
☐	☒	For null hypothesis testing, the test statistic (e.g. *F*, *t*, *r*) with confidence intervals, effect sizes, degrees of freedom and *P* value noted<br>*Give P values as exact values whenever suitable.*
☒	☐	For Bayesian analysis, information on the choice of priors and Markov chain Monte Carlo settings
☒	☐	For hierarchical and complex designs, identification of the appropriate level for tests and full reporting of outcomes
☐	☒	Estimates of effect sizes (e.g. Cohen's *d*, Pearson's *r*), indicating how they were calculated

Our web collection on statistics for biologists contains articles on many of the points above.

Software and code

Policy information about availability of computer code

Data collection	No new custom software was used for data collection.
Data analysis	The benchmarking experiment analysis code was provided in Code Ocean https://doi.org/10.24433/CO.4249201.v1, and detailed in the associated software policy file and readme.txt

For manuscripts utilizing custom algorithms or software that are central to the research but not yet described in published literature, software must be made available to editors and reviewers. We strongly encourage code deposition in a community repository (e.g. GitHub). See the Nature Portfolio guidelines for submitting code & software for further information.

Data

Policy information about availability of data

All manuscripts must include a data availability statement. This statement should provide the following information, where applicable:

- Accession codes, unique identifiers, or web links for publicly available datasets
- A description of any restrictions on data availability
- For clinical datasets or third party data, please ensure that the statement adheres to our policy

All data supporting the findings of this study are available within the paper and its Supplementary Information. The raw image data supporting the findings of this study are available on request due to their large sizes for uploading to public repositories. Source Data File has been provided for Fig 7f, g.

Research involving human participants, their data, or biological material

Policy information about studies with human participants or human data. See also policy information about sex, gender (identity/presentation), and sexual orientation and race, ethnicity and racism.

Reporting on sex and gender	Male, 77
Reporting on race, ethnicity, or other socially relevant groupings	Asian, Chinese.
Population characteristics	Single subject study for technology development.
Recruitment	Antemortem consent of donating post-mortem tissues.
Ethics oversight	Not applicable.

Note that full information on the approval of the study protocol must also be provided in the manuscript.

Field-specific reporting

Please select the one below that is the best fit for your research. If you are not sure, read the appropriate sections before making your selection.

☒ Life sciences ☐ Behavioural & social sciences ☐ Ecological, evolutionary & environmental sciences

For a reference copy of the document with all sections, see nature.com/documents/nr-reporting-summary-flat.pdf

Life sciences study design

All studies must disclose on these points even when the disclosure is negative.

Sample size	This is a method development paper, no sample size calculation was required for the data supporting the interpretation of the study.
Data exclusions	No data was excluded, failed antibodies were stated accordingly.
Replication	All experiments were replicated at least twice.
Randomization	Not applicable to the method development study here.
Blinding	Not applicable to the method development study here.

Reporting for specific materials, systems and methods

We require information from authors about some types of materials, experimental systems and methods used in many studies. Here, indicate whether each material, system or method listed is relevant to your study. If you are not sure if a list item applies to your research, read the appropriate section before selecting a response.

Materials & experimental systems

n/a	Involved in the study	
☐	☒	Antibodies
☒	☐	Eukaryotic cell lines
☒	☐	Palaeontology and archaeology
☐	☒	Animals and other organisms
☒	☐	Clinical data
☒	☐	Dual use research of concern
☒	☐	Plants

Methods

n/a	Involved in the study	
☒	☐	ChIP-seq
☒	☐	Flow cytometry
☐	☒	MRI-based neuroimaging

Antibodies

Antibodies used	As described in Methods section and Supplementary Table 2
Validation	Validation experimental details were stated in the Methods section, "Screening antibodies compatible with INSIHGT"

Animals and other research organisms

Policy information about studies involving animals; ARRIVE guidelines recommended for reporting animal research, and Sex and Gender in Research

Laboratory animals	C57BL/6 mice from age post-natal 1 day to 36 months. The mice were provided by the Laboratory Animal Service Center of CUHK and maintained at a controlled temperature (22–23 °C) with an alternating 12 h light–dark cycle with free access to standard mouse diet and water. The ambient humidity was maintained at <70% relative humidity.
Wild animals	None
Reporting on sex	This method development study was agnostic to the sex of the animal tissue used.
Field-collected samples	None.
Ethics oversight	All experimental procedures were approved by the Animal Research Ethics Committee of the Chinese University of Hong Kong (CUHK) and were performed in accordance with the Guide for the Care and Use of Laboratory Animals.

Note that full information on the approval of the study protocol must also be provided in the manuscript.

Plants

Seed stocks	*Report on the source of all seed stocks or other plant material used. If applicable, state the seed stock centre and catalogue number. If plant specimens were collected from the field, describe the collection location, date and sampling procedures.*
Novel plant genotypes	*Describe the methods by which all novel plant genotypes were produced. This includes those generated by transgenic approaches, gene editing, chemical/radiation-based mutagenesis and hybridization. For transgenic lines, describe the transformation method, the number of independent lines analyzed and the generation upon which experiments were performed. For gene-edited lines, describe the editor used, the endogenous sequence targeted for editing, the targeting guide RNA sequence (if applicable) and how the editor was applied.*
Authentication	*Describe any authentication procedures for each seed stock used or novel genotype generated. Describe any experiments used to assess the effect of a mutation and, where applicable, how potential secondary effects (e.g. second site T-DNA insertions, mosiacism, off-target gene editing) were examined.*

Magnetic resonance imaging

Experimental design

Design type	Ex vivo whole human brain imaging.
Design specifications	Imaging of 1 human brain embedded in 1% agarose for obtaining gross anatomy image.
Behavioral performance measures	Not applicable.

Acquisition

Imaging type(s)	Structural
Field strength	3T
Sequence & imaging parameters	A T2 spin echo sequence was used for acquiring the ex vivo human whole brain image, with compressed sensing SPACE MRI.
Area of acquisition	Whole brain.

Diffusion MRI ☐ Used ☒ Not used

Preprocessing

Preprocessing software	Not an MRI-based behavioural study, not applicable.
Normalization	Not an MRI-based behavioural study, not applicable.
Normalization template	Not an MRI-based behavioural study, not applicable.
Noise and artifact removal	Not done.
Volume censoring	Not done.

Statistical modeling & inference

Model type and settings	No statistical test was performed on the image registration demonstration.
Effect(s) tested	Not applicable.

Specify type of analysis: [x] Whole brain [ ] ROI-based [ ] Both

Statistic type for inference<br>(See Eklund et al. 2016)	Not applicable.
Correction	Not applied.

Models & analysis

n/a	Involved in the study
[x]	[ ] Functional and/or effective connectivity
[x]	[ ] Graph analysis
[x]	[ ] Multivariate modeling or predictive analysis
